# Cenozoic losses of tree diversity constrain climatic adaptation of Western and Central European forests

**DOI:** 10.1038/s41467-026-77806-4

**Published:** 2026-09-16

**Authors:** Vincent Wilkens, Anna Walentowitz, Sebastian Teichert, Lisa Hülsmann, Nicolai Nürk, Ole R. Vetaas, Carl Beierkuhnlein

**Affiliations:** 1 Department of Biogeography, University of Bayreuth, Bayreuth, Germany; 2 Bayreuth Center of Ecology and Environmental Science (BayCEER), Bayreuth, Germany; 3 GeoZentrum Nordbayern, Friedrich-Alexander-Universität Erlangen-Nürnberg (FAU), Erlangen, Germany; 4 Ecosystem Analysis and Simulation (EASI) Lab, University of Bayreuth, Bayreuth, Germany; 5 Plant Systematics, University of Bayreuth, Bayreuth, Germany; 6 Department of Geography, University of Bergen, Bergen, Norway; 7 Geographical Institute Bayreuth, GIB, Universitätsstraße 30, Bayreuth, Germany; 8 Departamento de Botánica, Campus Universitario de Fuentenueva 18071, University of Granada, Granada, Spain

**Keywords:** Palaeoecology, Climate-change impacts, Biodiversity, Forest ecology

## Abstract

Paleoclimatic changes during the late Cenozoic led to substantial losses of tree diversity in Western and Central Europe. A modern synthesis linking which taxa were lost, the timing of these losses, and their implications for the adaptation of the region’s forest ecosystems to ongoing climate change is needed. Here, we compile a dataset of fossil occurrences of tree genera from Western and Central Europe to analyze tree diversity changes during the Cenozoic (66 Ma – present). We find that tree genus richness declined 75% after its peak in the Miocene (23 – 5.3 Ma), with most losses already occurring during the Plio-Pleistocene transition (ca. 2 – 3 Ma). We then use genus-level niche models to explore how these changes unfolded within climate space. Our results show how the current regional tree flora was progressively filtered towards the colder and drier margins of climate space. This coincided with the losses of most heat-tolerant lineages, including many temperate genera that could have profited under modern climate warming (e.g., *Castanea*, *Juglans, Aesculus*). We foresee several ways in which our analyses of paleoecological data can inform forest management to increase regional tree diversity and enhance forest resilience under climate change.

## Abstract (web summary)

This study shows that European tree diversity significantly declined over the Cenozoic due to colder conditions and glaciations. The loss of many heat-tolerant species constrains the resilience of European forests to the warming climate.

## Introduction

Current patterns of taxonomic, functional, and phylogenetic diversity in trees (i.e., tree diversity) across the Holarctic realm have been shaped by paleoclimatic changes^1^. As global temperatures declined and polar ice sheets reformed during the late Cenozoic (66 Ma—present), temperate trees gradually succeeded subtropical and tropical trees at middle to high latitudes^2–5^. In Western and Central Europe, temperate tree diversity remained high leading up to the Plio-Pleistocene transition (2–3 Ma)^6,7^. However, over the Pleistocene, repeated glaciations triggered regional extinctions (i.e., extirpations) of many temperate tree taxa that persisted in other parts of the Holarctic realm^8^. The prevailing explanation is that east-west oriented geographic barriers, such as the European Alps or the Mediterranean Sea, impeded the south- and northward range shifts of trees in response to paleoclimatic changes^9–11^. Today, in contrast with temperate forests in North America and Eastern Asia, forests in Western and Central Europe are a global cold spot of tree diversity^12^. While several foundational reviews documenting changes in Cenozoic tree diversity exist^2,13^, a modern synthesis linking which taxa were lost, the timing of these losses, and their implications for the adaptation of Western and Central European forests to future climate is still missing.

Recent episodes of tree mortality linked to severe droughts^14^ and insect outbreaks^15,16^ highlight the widespread vulnerability of temperate European forests to ongoing climate change^17,18^. Post-disturbance reorganization of these forests towards more resilient assemblages will probably require changes in species composition^19^. However, the capacity for both managed and passive species turnover is limited by the pool of tree species (i.e., the “option-space”)^20^ expected to remain suitable under future climate scenarios. Although modeling studies have identified both climate change ‘winners’ and ‘losers’ among native tree species^21^, the option-space for forestry is forecasted to narrow substantially over the coming century^20^. This presents a complex problem for forest management in Western and Central Europe, potentially limiting the establishment of resilient, productive, and biodiverse forests using only native species. Considering that this problem has likely been exacerbated by tree diversity changes during the Cenozoic, a paleoecological perspective connecting past losses to current deficits is needed. While species-level identification of tree fossil data is typically not possible, such data can provide insights as to what tree genera were present in Western and Central Europe in the past.

**Fig. 1:**
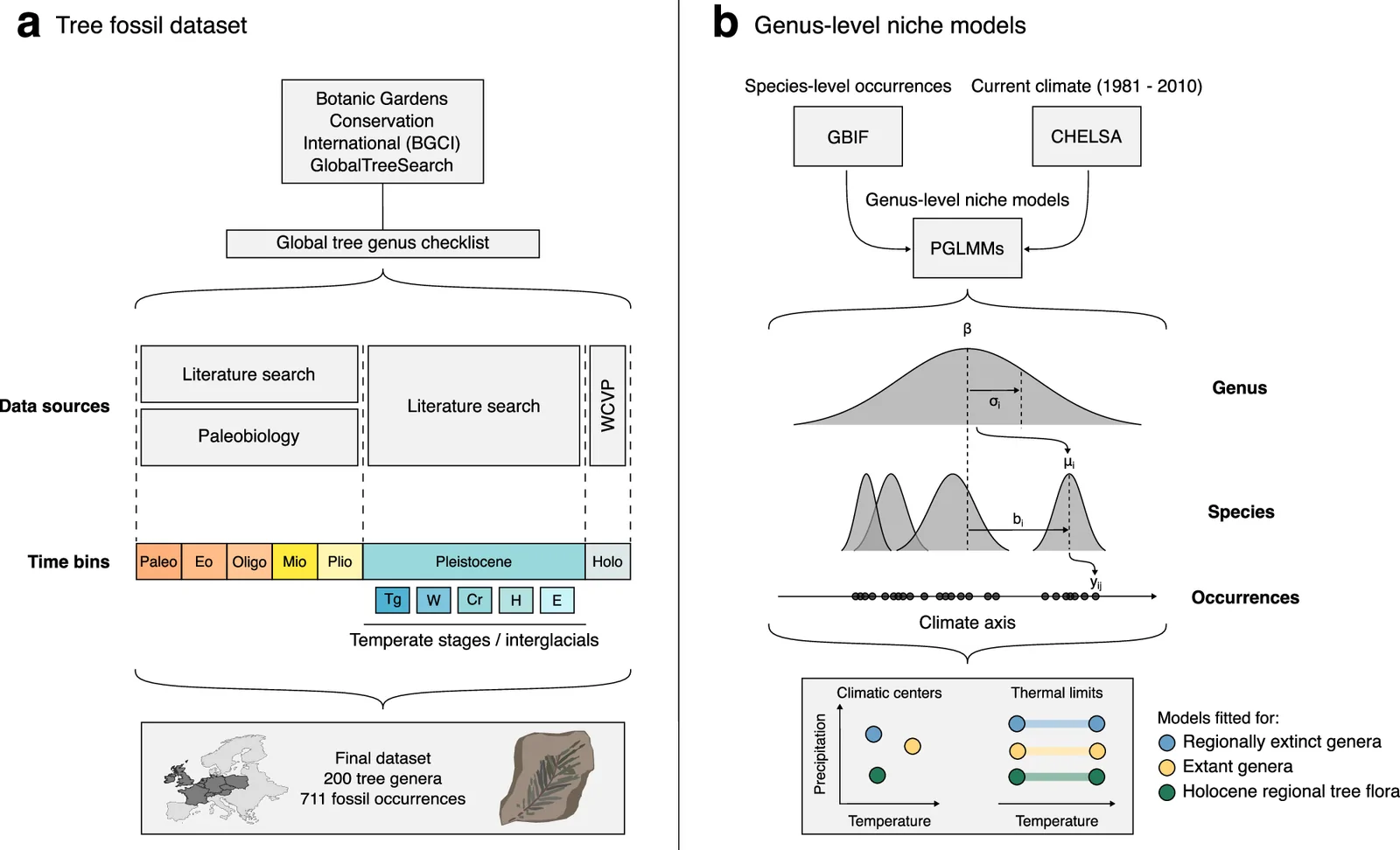
Overview of tree fossil dataset and genus-level niche models. **a** 711 fossil occurrences of tree genera during the Cenozoic (66 Ma – present) from Western and Central Europe (including Austria, Belgium, Czechia, France, Germany, Ireland, Luxembourg, Netherlands, Poland, Switzerland, and United Kingdom)^86^ were compiled using the Paleobiology Database (PBDB)^67^ and literature. Time bin abbreviations are as follows: Paleo – Paleocene, Eo – Eocene, Oligo – Oligocene, Mio – Miocene, Plio – Pliocene, and Holo – Holocene. For the Pleistocene, additional fossil occurrences dated to five temperate stages and interglacial periods were included: Tg – Tiglian, W – Waalian, Cr – Cromer, H – Holstein interglacial, and E – Eem interglacial. Holocene occurrences were obtained from the World Checklist of Vascular Plants (WCVP)^64^. **b** Species-level occurrence data from the Global Biodiversity Information Facility (GBIF) were combined with climate data from CHELSA (Climatologies at high resolution for the Earth’s land surface areas)^71,72^ to fit phylogenetic generalized linear mixed models (PGLMMs) of genus-level climatic centers and thermal limits. These models were used to estimate genus-level averages (β) of niche parameters while accounting for different sources of uncertainty. The fossil illustration in Fig. 1a was created by the lead author and is based on a photograph of a *Taxodium* fossil leaf by Amaltheus (available at https://commons.wikimedia.org/wiki/File:Fossil-leaf_Taxodium_dubium_Tertiary_Germany.jpg under a CC BY-SA 3.0 license).

Here, we contribute a paleoecological perspective on the dwindling option-space for forestry in Western and Central Europe under climate change. First, we compile a dataset of published fossil occurrences of tree genera (see Methods for definition) in Western and Central Europe to explore tree diversity changes throughout the Cenozoic (Fig. 1a). We also identify fossil occurrences dated to five temperate stages and interglacial periods within the Pleistocene, improving the temporal resolution of our dataset in the geologically recent past. Using this dataset, we ask (a) how tree diversity changed over the Cenozoic in Western and Central Europe and (b) which tree genera became regionally extinct during the Pleistocene.

Next, we aim to understand the impact of past tree diversity changes on the reorganizing capacity of Western and Central European forests amid ongoing climate change. We fit phylogenetic generalized linear mixed models (PGLMMs) of climatic centers and thermal limits for all tree genera that had a presence in Western and Central Europe during the Cenozoic (Fig. 1b). Models were fit using modern species occurrences and climate data (1981–2010) under a Bayesian framework to account for different sources of uncertainty (see Methods for detailed explanation). These hierarchical models are used to estimate genus-level averages of niche parameters while incorporating species-level phylogenetic covariance and correcting for sampling bias. We compare niche parameters among three analytical groups with respect to Western and Central Europe: (i) regionally extinct genera, (ii) extant genera, and (iii) the current option-space for forest reorganization, hereafter referred to as the “Holocene regional tree flora”. For regionally extinct and extant genera, niche parameters are estimated using all available tree species within each genus globally. For the Holocene regional tree flora, we fit separate niche models for each extant genus using only the native species pools of Western and Central Europe. We then examine all three groups in the context of current climate and future warming scenarios for Western and Central Europe. With this analysis, we ask (c) how tree diversity changes over the Cenozoic impacted the reorganizing capacity of current Western and Central European forests.

## Results

### Tree diversity changes during the Cenozoic

We started by summarizing major trends in tree genus richness during the Cenozoic based on fossil occurrences, highlighting differences between angiosperms and gymnosperms (i.e., conifers and *Gingko*). For all analyses shown, we defined a tree genus as having at least 30% of its accepted species recognized as tree-forming (see Methods for details).

**Fig. 2:**
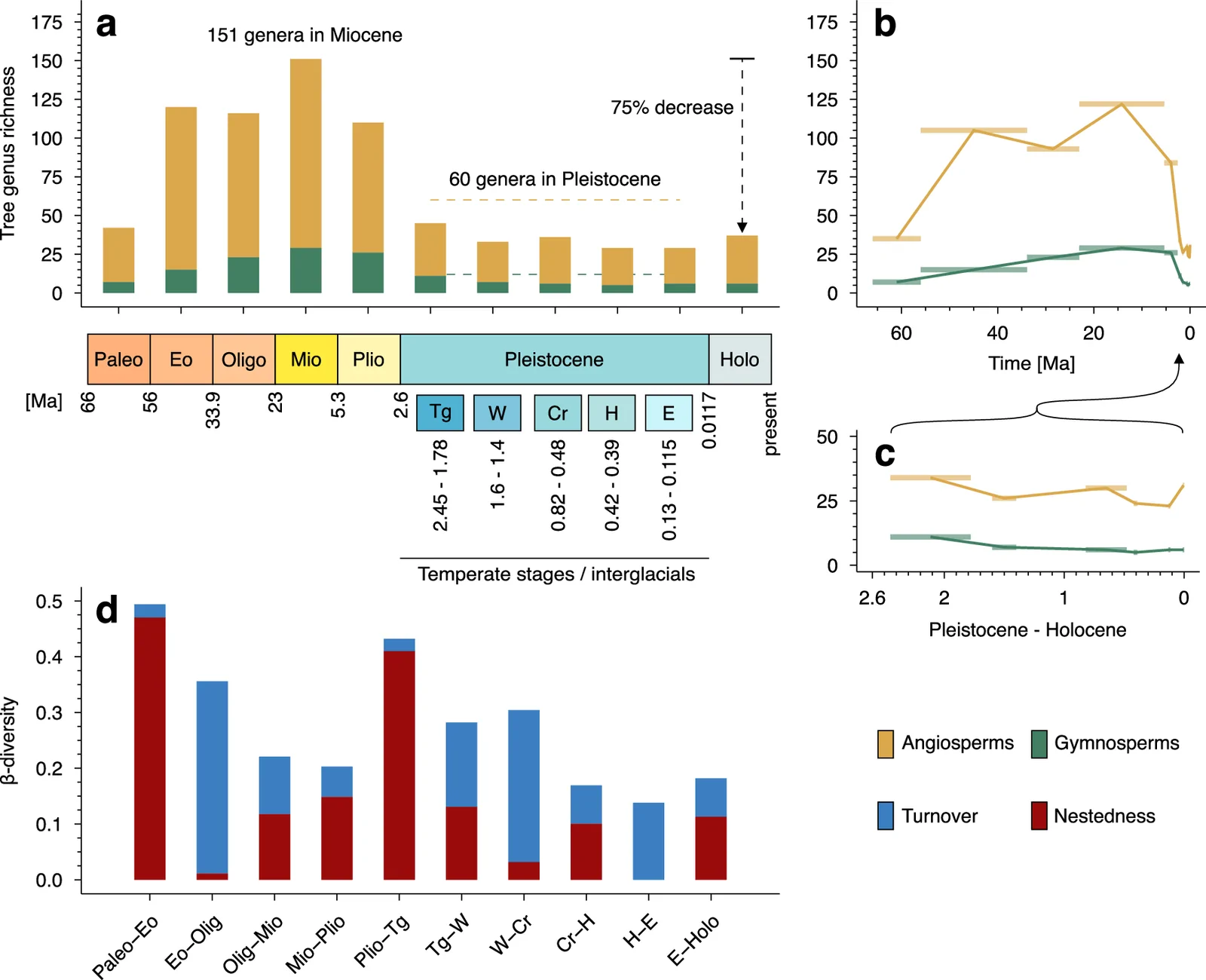
Tree genus diversity changes in Western and Central Europe over the Cenozoic (66 Ma – present) based on compiled fossil occurrences. **a** Tree genus richness changes over 11 time bins, highlighting differences between angiosperms (orange) and gymnosperms (green). Time bin abbreviations are as follows: Paleo – Paleocene, Eo – Eocene, Oligo – Oligocene, Mio – Miocene, Plio – Pliocene, and Holo – Holocene. For the Pleistocene, additional fossil occurrences dated to five temperate stages and interglacial periods were included: Tg – Tiglian, W – Waalian, Cr – Cromer, H – Holstein interglacial, and E – Eem interglacial. **b** Tree genus richness changes on a linear timescale. Lines connect the midpoints of each time bin from (**a**), with a closer look at the Pleistocene-Holocene interval (2.6 – present) in (**c**). **d** Total ß-diversity changes between consecutive time bins, broken down into nestedness (red) and turnover (blue) components.

Compiled fossil data from Western and Central Europe showed a steady increase in tree genus richness throughout most of the Cenozoic, followed by a significant decline after the Miocene (Fig. 2a, b). This increase was interrupted only by a small decrease over the Eocene-Oligocene boundary, during which angiosperms (11% decrease) and gymnosperms (53% increase) responded differently. In the Miocene, tree genus richness reached an overall peak of 151 genera, including 122 angiosperms and 29 gymnosperms. Tree genus richness subsequently decreased 27% in the Pliocene, followed by a further 59% decrease in the Tiglian stage of the Early Pleistocene. During the Pleistocene, tree genus richness remained comparatively stable, but reached an overall low of 29 genera in the Holstein and Eem interglacials (Fig. 2a, c). When taken as a whole, Western and Central Europe hosted 60 tree genera in the Pleistocene (45% decrease from the Pliocene). Of the 200 tree genera found in the fossil record of Western and Central Europe during the Cenozoic, only 37 have a native presence in the region today. Altogether, tree genus richness decreased 75% from its peak in the Miocene to the Holocene, with gymnosperms (79% decrease) more impacted than angiosperms (75% decrease). We conducted sensitivity analyses to evaluate how varying the minimum threshold for classifying a genus as a tree genus, defined by the proportion of species within the genus known to be tree-forming, affects these findings (Supplementary Figs. 1–3). These analyses confirmed the robustness of all trends, though the absolute numbers of genera varied.

Next, we calculated turnover (ß_TURN_) and nestedness (ß_NEST_) components of ß-diversity (ß_TOTAL_) between all consecutive time bins using the Sørensen dissimilarity index (Fig. 2d). Total ß-diversity exhibited two peaks during the Cenozoic, both driven primarily by the nestedness component, reflecting large losses or gains of genera with few replacements. The first peak was the Paleocene-Eocene transition (ß_TOTAL_ = 0.494, ß_TURN_ = 0.024, ß_NEST_ = 0.470), which coincided with the emergence of 78 genera in the fossil record. The second peak was the Pliocene-Tiglian transition (ß_TOTAL_ = 0.432, ß_TURN_ = 0.022, ß_NEST_ = 0.410), over which 65 genera disappeared from Western and Central Europe (52 permanently). During the Eocene-Oligocene transition (ß_TOTAL_ = 0.356, ß_TURN_ = 0.345, ß_NEST_ = 0.011), turnover was the dominant component, indicating that changes in total ß-diversity resulted largely from genera being replaced by others. This timespan was marked by the first appearances of many temperate tree genera (e.g., *Picea*, *Betula*, *Fagus*) as well as the first losses of tropical tree genera, including palms (e.g., *Areca*, *Livistona*, *Serenoa*) and mangroves (e.g., *Nypa*, *Xylocarpus*, *Excoecaria*). During the Pleistocene, total ß-diversity trended downwards. However, absolute gains and losses of tree genera were small, and compositional changes were strongly influenced by intermittent gaps in the fossil record. Sensitivity analyses showed that both trends and absolute changes in ß-diversity were not dependent on the definition of a tree genus, with some breakdown observed at the most conservative threshold proportion of 1.0, which is attributable to the limited numbers of genera meeting this criterion (Supplementary Figs. 4–6).

**Fig. 3:**
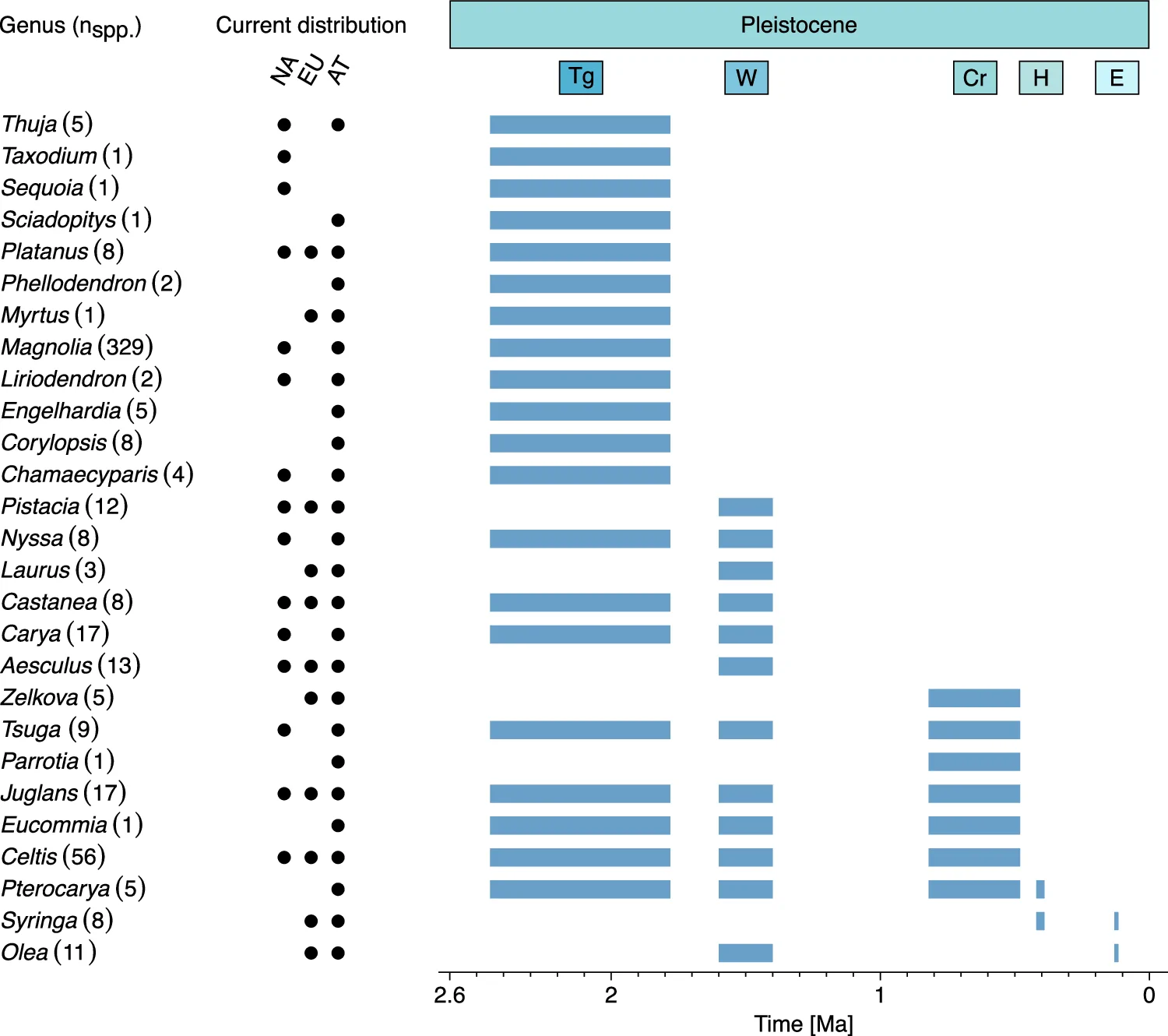
All regionally extinct tree genera in Western and Central Europe (WCE) that disappeared during the Pleistocene. The number of accepted tree species according to Botanic Gardens Conservation International (BGCI) GlobalTreeSearch database are given^63^. Current distribution in NA: North America, EU: Europe (excluding WCE), and AT: temperate Asia based on continental distribution data from World Checklist of Vascular Plants^64^. Pleistocene interglacials and temperate stages are abbreviated as follows: Tg Tiglian, W Waalian, Cr Cromer, HHolstein interglacial, and E Eem interglacial.

Most regional extinctions of tree genera in Western and Central Europe took place during the Plio-Pleistocene transition or earlier (Fig. 2a). Compiled fossil data revealed only 27 tree genera that went regionally extinct during the Pleistocene (Fig. 3). During the Tiglian stage, 12 tree genera were lost, including several currently found in temperate forests of North America and Eastern Asia, such as redwood (*Sequoia*), arborvitae (*Thuja*), and swamp cypress (*Taxodium*). Certain temperate genera, including hemlock (*Tsuga*) or hickory (*Carya*), maintained a presence into the Waalian or Cromer stage, respectively. Other genera that disappeared from Western and Central Europe during the Middle Pleistocene, such as chestnut (*Castanea*), horse chestnut (*Aesculus*), walnut (*Juglans*), laurel (*Laurus*), and pistachio (*Pistacia*), maintain native populations in the Mediterranean basin today. From the Holstein interglacial to the Holocene, only three additional genera were lost: olive (*Olea*), wingnut (*Pterocarya*), and lilac (*Syringa*).

### Linking past losses to current deficits in forest reorganization

**Fig. 4:**
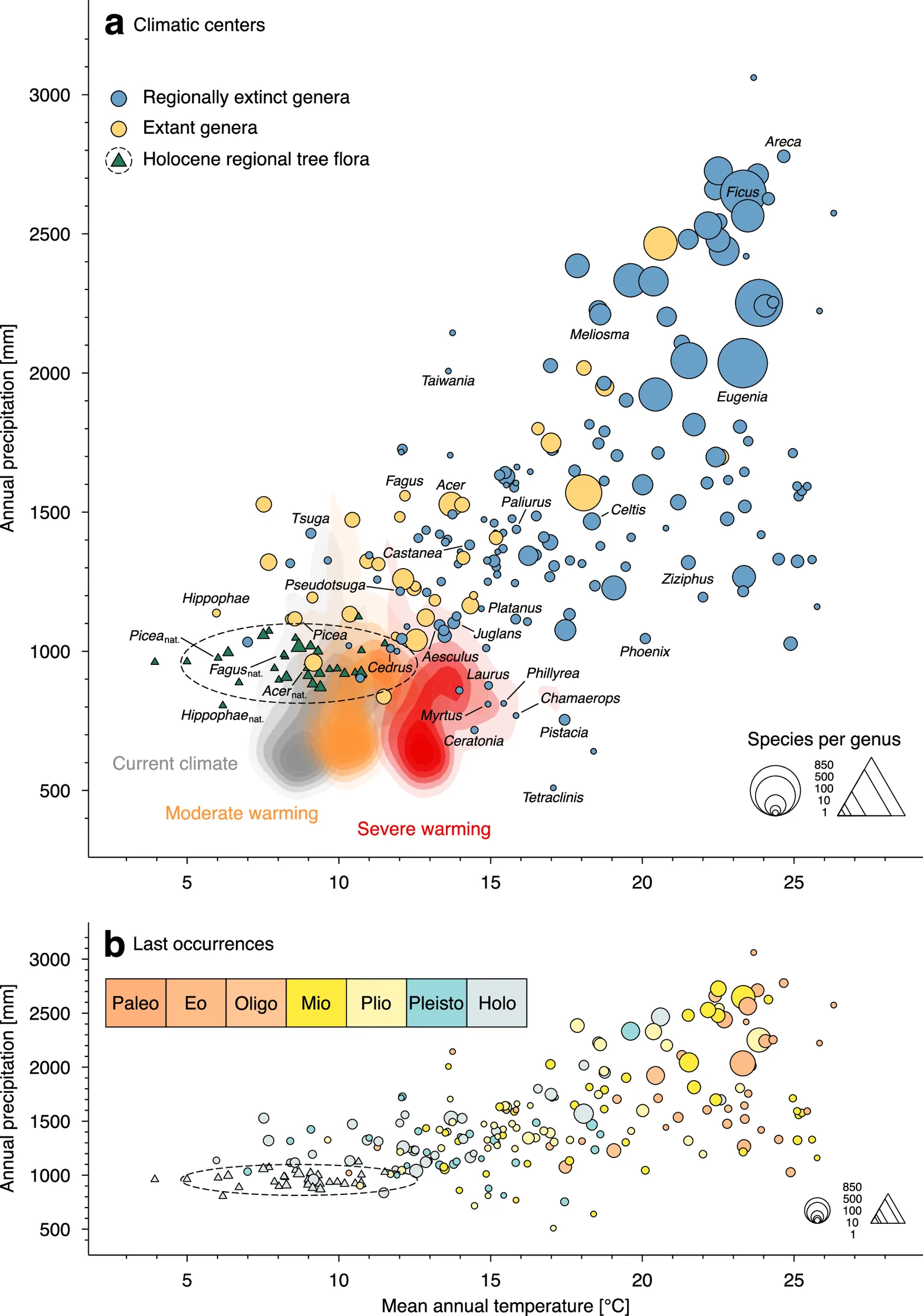
Average locations in climate space of Cenozoic tree genera from Western and Central Europe. **a** Estimated climatic centers of 200 tree genera, comparing (i) regionally extinct genera (blue), (ii) extant genera (yellow), and (iii) the current option-space for forest reorganization, referred to as the Holocene regional tree flora (green). Climatic centers of regionally extinct and extant genera were estimated using all available tree species within each genus globally, whereas climatic centers of the Holocene regional tree flora were estimated for each extant genus using only the native species pools of Western and Central Europe (e.g., *Abies*_nat._ represents *Abies alba* Mill.). The Gelman-Rubin criterion ($\hat{R}$) was used to assess Bayesian model convergence, with values closer to 1.0 indicating successful convergence. Altogether, of the 232 models fitted, five were excluded from the plot because of convergence problems ($\hat{R}$ > 1.05 or number of divergent transitions > 40), including *Salix, Eucalyptus*, *Prunus*, *Zanthoxylum*, and *Betula*_nat_. Density clouds of current and future climate scenarios have been underlaid^71,72^. Current climate: 1981–2010. Moderate warming: SSP1-2.6 ( + 1.3 °C–2.4 °C by 2100). Severe warming: SSP5-8.5 ( + 3.3 °C–5.7 °C by 2100). **b** Estimated climatic centers of 200 tree genera (same as in **a**), with colors corresponding to the timing of the last known fossil occurrence of each genus in Western and Central Europe. **a**,**b** Point size scales with the number of accepted tree species within each genus.

We used PGLMMs to determine the average locations of Cenozoic tree genera within a bivariate climate space of mean annual temperature and annual precipitation (Fig. 4a). Our results show how most extant genera as well as the Holocene regional tree flora are distinctly clustered towards the colder and drier margins of climate space (e.g., *Acer*, *Fagus*, *Picea* in Fig. 4a). Incorporating the last known fossil occurrence of each genus from Western and Central Europe further revealed that this clustering is linked to a strong directional pattern of filtering across time (Fig. 4b). In contrast, most regionally extinct genera (e.g., *Areca*, *Meliosoma*, *Eugenia*, *Ziziphus*, *Phoenix* in Fig. 4a) occupy much warmer and wetter conditions. Regionally extinct genera also collectively span the broadest range in climatic conditions, including tropical, subtropical, and temperate climates, but are more absent towards the colder and drier margins. Compared with extant genera, corresponding genera in the Holocene regional tree flora (representing only native species from Western and Central Europe) show a general shift towards, on average, colder and drier locations in climate space (e.g., compare *Acer* and *Acer*_nat_ in Fig. 4a). Both regionally extinct and extant genera are evenly distributed across temperate parts of climate space.

**Fig. 5:**
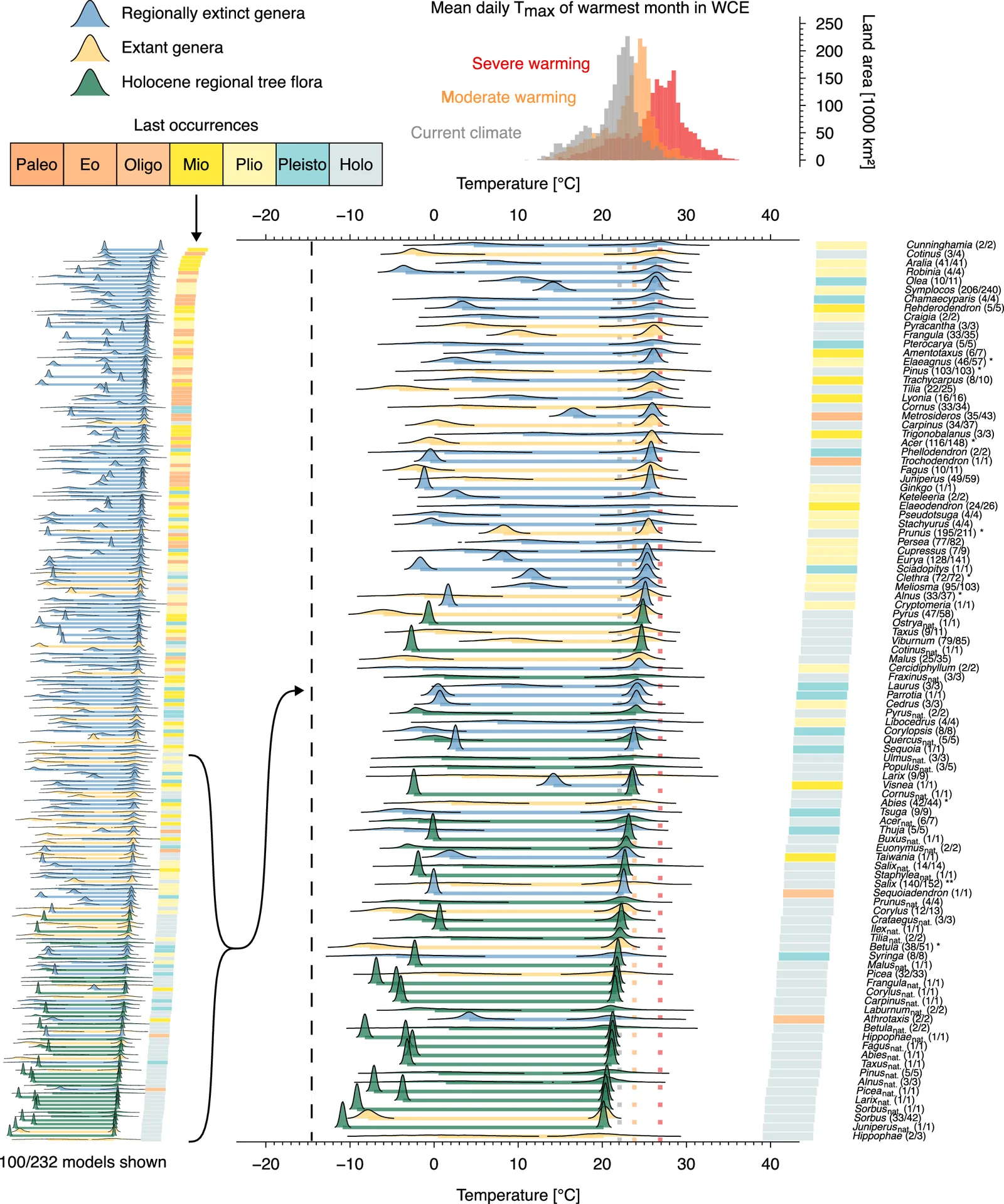
Upper and lower thermal limits of Cenozoic tree genera from Western and Central Europe. Posteriors of upper and lower thermal limits for 200 tree genera were estimated, comparing (i) regionally extinct genera (blue), (ii) extant genera (yellow), and (iii) the current option-space for forest reorganization, referred to as the Holocene regional tree flora (green). Thermal limits of regionally extinct and extant genera were estimated using all available tree species within each genus globally, whereas thermal limits of the Holocene regional tree flora were estimated for each extant genus using only the native species pools of Western and Central Europe. Models were arranged by increasing upper thermal limit (bottom to top). Only the first 100 of 232 models are shown (see Supplementary Fig. 7 for full figure including all models). Color stripes correspond to the last known occurrence of each genus in the fossil record of Western and Central Europe. All genera have been labeled on the right-hand side of the plot, including the number of species from all accepted species in each genus used to fit the models. The Gelman-Rubin criterion ($\hat{R}$) was used to assess Bayesian model convergence, with values closer to 1.0 indicating successful convergence. Genera with $\hat{R}$ > 1.01 (*) or $\hat{R}$ > 1.05 (**) were labeled as having potentially unreliable estimates. The distribution of heat extremes (mean daily maximum temperature of the warmest month) by land area under current and future climate scenarios in Western and Central Europe is overlaid^71,72^. Current climate: 1981–2010. Moderate warming: SSP1-2.6 ( + 1.3 °C–2.4 °C by 2100). Severe warming: SSP5-8.5 ( + 3.3 °C–5.7 °C by 2100).

To compare the thermal tolerances of Cenozoic tree genera, we used PGLMMs to estimate upper and lower thermal limits, defined here as the genus-level averages of mean daily maximum temperature of the warmest month and mean daily minimum temperature of the coldest month, respectively (Fig. 5; see Supplementary Fig. 7 for full figure). Genera were arranged by increasing upper thermal limit (bottom to top), since this niche parameter will be relevant under ongoing climate warming. Overall, the Holocene regional tree flora exhibits the lowest tolerances for on average higher temperatures compared to almost all extant and regionally extinct genera. The Holocene regional tree flora also exhibits the greatest cold tolerances, though many regionally extinct and extant genera show similar capacity to tolerate on average colder temperatures while still maintaining high upper thermal limits (e.g., *Robinia* or *Tilia* in Fig. 5). Compared to the Holocene regional tree flora, corresponding genera within extant genera show much higher upper thermal limits (e.g., compare *Pinus* and *Pinus*_nat_ in Fig. 5). Future warming scenarios in Western and Central Europe will shift heat extremes further towards these upper limits (Fig. 5). Furthermore, including the last known fossil occurrence of each genus reveals that most genera with high thermal limits disappeared from Western and Central Europe, particularly from the Miocene onwards (Fig. 5).

## Discussion

This study combined fossil data with climatic niche models to provide an overview of past tree diversity changes in Western and Central Europe as well as to explore how these changes unfolded within climate space. Our results highlight current deficits in regional tree diversity and potential limitations in the capacity of forests to reorganize amid climate warming, linking these to paleoecological legacies during the late Cenozoic. We demonstrate how insights from the past 66 million years can improve our understanding of current forest ecosystems and guide management strategies to adapt forests to future climate.

Using our fossil dataset, we give a detailed account of the substantial decline in tree genus richness in Western and Central Europe after the Miocene (23–5.3 Ma). We found that most losses took place before the Tiglian stage of the Early Pleistocene ( > 2.45 Ma), with a few regionally extinct tree genera even persisting into the Middle Pleistocene (ca. 820–115 ka). European tree diversity has been recognized as lower than other continents for a long time^7,9,12,22^. Unlike previous studies, we focus on Western and Central Europe, which encompasses a major portion of managed temperate forest ecosystems in Europe today. Our results indicate that this region was especially impacted by late Cenozoic extinctions, as evidenced by the regional losses of tree genera (e.g., *Castanea*, *Juglans*, *Aesculus*) that have persisted in other parts of Europe. Furthermore, our research synthesizes previous global^12,22^ and regional^2,7^ studies to provide a continuous, high-resolution overview of tree diversity changes in Western and Central Europe for the entire Cenozoic. This includes five time bins in the Pleistocene that improve coverage when both paleoclimatic variability and extinctions were at their highest. The compiled dataset is freely available and can form the basis for further biogeographical studies (see Data Availability).

Past regional extinctions of tree genera in Western and Central Europe coincided with a strong shift in favor of lineages adapted to colder and drier climates. These results are consistent with previous analyses^7,22^ as well as reconstructed paleoclimatic cooling and drying since the mid-Cenozoic^5^. In the process, most heat-adapted lineages were also found to have been progressively filtered from the regional tree flora. Filtering favored extant genera over regionally extinct genera in addition to more cold-adapted and drought-tolerant species within extant genera. Thus, our results show how past tree diversity changes in Western and Central Europe can be linked to projected limitations in the capacity of the current regional tree flora to respond to ongoing climate warming^20,21^. However, we note that our analysis does not indicate regionally extinct genera exhibiting clear advantages over extant genera under future climate scenarios. This suggests that lost genus-level diversity itself might not be a major barrier to forest reorganization. Instead, the lack of native species within extant genera, which would be suited to warmer and drier conditions in the future^23^, will be the main challenge for regional forest management. While extant genera contain such species globally in other temperate zones, they are missing in Western and Central Europe. Given these limitations, ongoing climate warming also risks inducing interaction effects with paleoclimatic cooling that greatly amplifies future losses in regional tree diversity^24^.

“Form genera” and other globally extinct taxa (e.g., *Laurophyllum*) were excluded from our analysis (see Methods). Consequently, estimated tree diversity losses likely represent a lower bound to true losses. If these unresolved lineages could be accounted for, the degree of regional extinctions would probably be even higher and could also shift drivers of early Cenozoic beta diversity from nestedness to turnover^25^. Next, we developed genus-level niche models to link paleoecological findings with contemporary biodiversity research and forest management^26^. These models leverage phylogenetic relationships within genera to account for the non-independence of occurrences and species as well as compensate for species-level differences in occurrence data availability. A necessary assumption underlying this approach is that closely related species will tend to occupy similar environmental niches (i.e., phylogenetic niche conservatism). This assumption is well-founded^7,22,27–31^ and is used frequently to reconstruct paleoclimate^32–35^. If phylogenies have not been adequately resolved, estimates for individual genera could be biased, but this would not impact the overall interpretation of our findings. We also counteracted sampling biases in species occurrence data by weighting the likelihood contribution of each occurrence as proportional to the sampling effort required to obtain it (see Methods)^36^. This approach has previously been shown to help recover unbiased regression coefficients^37^. Furthermore, correlative methods produce models of the realized niche, which could lead to underestimation of thermal limits when abiotic and biotic constraints on the fundamental niche are not accounted for^38^. We assumed these issues would average out when modeling niches at the genus level, particularly when drawing on statistical power from many species. While our models are more robust because of this, they are less interpretable since they represent genus-level averages, whereas individual species can deviate significantly from these averages.

Forest reorganization in Western and Central Europe will require shifts towards more heat-adapted and drought-tolerant species in accordance with increasing temperatures and precipitation variability^39–41^. If compositional changes are disrupted, such as when current stocks of young trees face high cultivation risk^42^, forests could degrade into alternative ecosystem states with major implications for the global carbon sink, local biodiversity, and ecosystem services^43,44^. Our findings highlight limitations in the capacity of current Western and Central European forest ecosystems to reorganize under ongoing climate warming, supporting the case for introducing non-native species to partially restore lost tree diversity and enhance climate resilience. However, the ecological risks associated with these measures are unpredictable and could threaten native biodiversity^45^. Even if not invasive themselves, non-native species can act as vectors for the spread of pests and diseases. Nevertheless, non-native tree species have played important roles in forestry, arboriculture, and urban planning in Europe for centuries, with instances of both successful establishment (*Robinia pseudoacacia* L.) and complete failure (*Pinus strobus* L.)^46,47^. Non-native tree species currently make up around 4% of European forest area (Brus et al., 2019), attributable to advantages in productivity and resilience over native species (e.g., *Pseudotsuga menziesii* Mirb., which is not prone to spruce bark beetle outbreaks)^48^.

Non-native species carry substantial uncertainty regarding their population trends and dominance patterns, but these risks could be mitigated with measures that incorporate insights from paleoecological data^49–52^. In particular, genera that were present in Western and Central Europe during the Pleistocene but persisted into the Holocene only in neighboring regions could be promoted using assisted migrations (e.g., broadleaved-deciduous species from Southern Europe like *Juglans regia* L. or *Castanea sativa* Mill.)^53^. The European biogeographical histories of some of these genera are still unresolved, as millennia of human cultivation, including in Western and Central Europe, obscure their natural post-glacial distributions^54–56^. Other Mediterranean genera, like *Laurus* or *Pistacia*, which we considered non-native to Western and Central Europe in our study, could become more prominent in the future as phytoclimatic shifts increasingly favor evergreen trees over needleleaf and cold-deciduous trees^57^. Considering these nuances, tree genera from neighboring regions warrant special consideration under forest reorganization in contrast to non-native genera from other continents like *Pseudotsuga*, which was introduced into the region only in the last two centuries^48^

Critically, the species represented in the fossil record by regionally extinct genera are probably not the same species that persist elsewhere today. Some species from regionally extinct genera with relictual populations in the Mediterranean basin may be an exception to this (e.g., *Aesculus hippocastanum* L.)^58^. Even if their modern relatives are suitable for cultivation, current forest ecosystems and their biotic interactions differ significantly from those of the Pleistocene, especially due to the absence of large herbivores^59^. Therefore, further research is needed to fill gaps about how lost genus-level diversity can be compensated for with non-native species given the invasiveness risks and high species-level variation within many genera. Extant genera also contain many non-native species from other continents or neighboring regions that will be better suited to future climate, have the potential to hybridize with native species, and may carry fewer risks since these genera have persisted in the region throughout the Cenozoic^60^. Furthermore, native tree diversity in Western and Central Europe is still underutilized as most forests are species-poor due to past and ongoing management rather than climatic constraints. Attempting to utilize the full option-space of native tree species diversity including genetic variation^61^ should be a priority alongside any alternative measures.

In summary, we document substantial losses of tree diversity in Western and Central Europe during the late Cenozoic accompanied by strong paleoclimatic filtering that has shaped the regional tree flora over tens of millions of years. These insights show that current challenges in regional forest management are deeply entrenched in paleoecological legacies, possibly requiring significant human intervention in forest ecosystems. In the end, striking a balance between the concerns of foresters and ecologists to increase regional tree diversity will be critical to forest reorganization.

## Methods

All data analyses were conducted in R v.4.4.2. (R Core Team, 2024).

### Tree diversity changes during the Cenozoic

#### Obtaining fossil data

To analyze how tree diversity changed throughout the Cenozoic era in Western and Central Europe, we first compiled a dataset of fossil occurrences of tree genera. We conducted our study at the genus level because species-level identification of tree fossils is generally not possible. This is due to critical identifying features being poorly preserved or the specimens not matching any known extant species. Our dataset spanned 11 time bins, corresponding to seven geological epochs in the Cenozoic era^62^. The Pleistocene was further divided into five temperate stages and interglacial periods, following the historical nomenclature from Lang^6^. The steps taken to compile the dataset are described in detail below.

We obtained a global checklist of tree species from the Botanic Gardens Conservation International GlobalTreeSearch database (BGCI; accessed 24/10/2024)^63^, which defines a tree in accordance with the IUCN Global Tree Specialist Group definition of “a woody plant with usually a single stem growing to a height of at least 2 meters, or if multi-stemmed, then at least one vertical stem five centimeters in diameter at breast height”. We standardized this checklist by matching all species names to the World Checklist for Vascular Plants (WCVP)^64^ using the R package “U.Taxonstand”^65,66^. Species for which a perfect match could not be found were removed.

Next, to establish a robust definition of a tree genus, we first calculated the proportion of tree species within each genus. We initially explored two approaches: either classifying a genus as a tree genus if it contained at least one tree-forming species or if it consisted entirely of tree-forming species. However, we noticed that the first approach tended to include many genera consisting predominantly of herbaceous or shrubby species, only a few of which are known to be tree-forming (e.g., *Erica*, *Vaccinium*, *Chenopodium*), while the second approach inadvertently excluded many widely recognized tree genera (e.g., *Salix*, *Quercus*, *Pinus*) since they also contain shrubby species. To address this, we conducted sensitivity analyses (Supplementary Figs. 1–6) to evaluate the impact of different thresholds on downstream analyses. Based on these results, we selected a threshold of 0.3 (at least 30% of species should be tree-forming) for all further analyses. By applying this threshold to our global checklist of tree species, we derived a global checklist of 3,354 tree genera.

We downloaded fossil occurrence data for Europe from the Paleobiology Database (PBDB; accessed 12/02/2026; see Data Availability)^67^. PBDB is a paleontological database that was chosen as the basis for our fossil dataset because it is curated and contains standardized fossil occurrences across all geological ages. However, because PBDB is incomplete, database occurrences from PBDB were supplemented with additional records identified through literature searches (search string: e.g., “*Quercus*” AND “fossil”) in Web of Science and Google Scholar. We focused mainly on peer-reviewed literature and accepted a study if the biostratigraphy or dating methods were explicitly described in the text or if it was a review-type article. We thoroughly scanned all studies for additional fossil occurrences, including occurrences of genera not contained within the PBDB dataset, resulting in 34 additional tree genera. We did not constrain this literature search to Europe, as studies from other continents often proved to be reliable sources for thorough reviews of the global fossil records of certain genera or families.

All occurrences were filtered to only include fossils dated to the Cenozoic era and which had been assigned to genera in our global tree genus checklist. “Form genera” and other globally extinct taxa were also excluded, as these cannot be reliably resolved to a modern genus. Furthermore, since we aim to link paleoecological filtering in deep time to current challenges in forest reorganization, it was conceptually necessary to focus on lineages that are not globally extinct. Next, we filtered occurrences to only include fossils from Western and Central Europe, defined in this study to encompass the following countries: Austria, Belgium, Czechia, France, Germany, Ireland, Luxembourg, Netherlands, Poland, Switzerland, and the United Kingdom. This study area was chosen because it forms the core of the European temperate zone, contains heavily managed forest ecosystems, and has been disproportionally impacted by paleoclimatic changes due to its geographic configuration.

Because the dating of Pleistocene occurrences within PBDB was too coarse to assign these to a specific interglacial period or temperate phase based on the chronology from Lang (1994)^6^, we excluded these from the PBDB dataset. Instead, occurrence data for the five temperate stages and interglacial periods in the Pleistocene were acquired exclusively through literature, which included several major monographs on the vegetation history of Europe^2,6^. The final fossil dataset comprised 711 occurrences from PBDB and literature, representing 200 tree genera.

Lastly, to determine the natural presence of genera in Western and Central Europe during the Holocene, we did not use fossil occurrences due to the scarcity of findings in PBDB as well as the difficulty of excluding human influences. Instead, we obtained a modern checklist of accepted native tree genera for Western and Central Europe using WCVP, accessed via the “rWCVP” R package^68^. WCVP determines a species to be native to an area where “[it] has been present since before the last Ice Age or arrived by natural colonization”^69^. We set an additional criterion for this checklist that excluded any genera if they were only native to France. This was done to avoid several Mediterranean genera from Southern France, which are atypical for temperate forest ecosystems of Western and Central Europe, from being classified as native to the region as a whole. These genera were *Juglans*, *Celtis*, *Paliurus*, *Laurus*, *Pistacia*, *Myrtus*, *Chamaerops*, *Phillyrea*, and *Ceratonia*. Furthermore, tree genera were only included for the Holocene if they had previously occurred in the fossil record of Western and Central Europe. This was done to rule out the possibility that new genera (without a previous presence during the Cenozoic) could have emerged during the Holocene, reducing biases inherent to comparing fossil occurrence data with modern country-level botanical checklists.

#### Analyzing tree diversity changes

We analyzed changes in tree genus richness and beta diversity throughout the Cenozoic era based on compiled fossil data. Tree genus richness was calculated based on the number of unique tree genera recorded within each time bin. Total ß-diversity as well as its nestedness and turnover components were calculated for all consecutive time bins in our fossil dataset using the Sørensen dissimilarity index with the “betapart” R package^70^. Compositional changes driven by turnover result from the replacement of genera by others, whereas changes driven by nestedness occur when genera are gained or lost.

### Building niche models of tree genera

#### Climate data

Global climate data were obtained from CHELSA (Climatologies at high resolution for the Earth’s land surface areas)^71,72^. We used the high-resolution climatologies based on GFDL-ESM4 (GFDL Earth System Model Version 4.1), following recommendations of the ISIMIP3b protocol. Climate data were resampled from 30″ to a 0.25° spatial resolution to reduce sampling bias, spatial autocorrelation, as well as the computational cost of model fitting.

#### Species occurrence data

Species occurrence data were downloaded from the Global Biodiversity Information Facility (GBIF)^73^ for all tree species belonging to genera in the final fossil occurrence dataset (*n* = 9407 species). Only occurrences with the following basis of record were included: “OBSERVATION”, “OCCURRENCE”, “HUMAN_OBSERVATION”, “PRESERVED_SPECIMEN”. Occurrence data were filtered to remove entries with missing coordinates, erroneously recorded coordinates, and coordinates with high GPS uncertainty ( > 10 km). Setting the GPS uncertainty threshold to 10 km also allowed us to accommodate both gridded occurrence datasets, like national forest inventories, as well as herbarium samples without exact geolocations^74^. Remaining occurrence data for each species were then spatially thinned to the same 0.25° resolution grid as the climate data using the “GeoThinneR” R package^75^. We used the “terra” R package^76^ to extract climate data at each species occurrence point.

#### Accounting for sampling bias

Geographical biases in species occurrence data present a major challenge for environmental niche modeling. Sampling efforts that generate this data tend to be heterogeneously distributed due to differences in human accessibility across landscapes. We counteracted this bias by weighting the likelihood contribution of each species occurrence point in proportion to the estimated sampling effort required to obtain that point. We used the “sampbias” R package^36^ to calculate weights based on a spatial projection of the estimated sampling rate across all species occurrence points. For this, we used the default gazetteers for cities, airports, roads, and rivers as well as the same 0.25° grid from climate and occurrence data.

#### Model fitting

Following the recommendations of Smith et al.^26^ for estimating niches at taxonomic levels above species, we fitted phylogenetic generalized linear mixed models (PGLMMs) to infer climatic centers and thermal limits for 200 tree genera. We distinguished between regionally extinct (*n* = 163) and extant genera (*n* = 37) in our analysis. Additionally, for each extant genus, we fitted PGLMMs using only the species native to Western and Central Europe, which we refer to as the Holocene regional tree flora (*n* = 37). We attempted to fit all 237 PGLMMs, but there were five cases where it was not possible to fit a model because the corresponding species pool contained no tree species. This resulted in a final total of 232 models of both climatic centers and thermal limits, respectively.

There are several critical advantages to using PGLMMs to fit genus-level niche models over alternative approaches^26^. First, we can directly account for both the statistical non-independence of occurrence data within species as well as the phylogenetic non-independence of species within genera^77^. With GLMs, occurrences across species are assumed to be statistically independent, which severely biases the inference of genus-level niches towards species that are overrepresented in the data (e.g., *Pinus sylvestris* in *Pinus*). While GLMMs account for statistical non-independence of occurrences, they still assume that species within a genus are statistically independent from one another. PGLMMs overcome these problems by incorporating shared evolutionary history of species through partial pooling, which is a feature of hierarchical models that enables information sharing across grouping variables^78^. In PGLMMs, the degree of information sharing is inversely proportional to the phylogenetic distance between species, which is regulated by a phylogenetic variance-covariance matrix. As a result, species-level parameters are adjusted toward genus-level estimates in a way that reflects shared evolutionary history and ecological limits within genera. Furthermore, this approach also improves the reliability of parameter estimates for rare species, compensating for sparse occurrence data by borrowing strength from common species through shared evolutionary relationships^79,80^.

We calculated the phylogenetic variance-covariance matrix (${{{\bf{A}}}}$) of each genus with the “ape” R package^81^. Phylogenies were generated with “U.PhyloMaker” using the “GBOTB.extended.WCVP” mega tree^65,82–84^. Species that were not included in the mega tree were bound to the basal node of each genus as polytomies.

The models were specified as intercept-only PGLMMs, including a genus-level fixed intercept and species-level random intercept with phylogenetic covariance structure. If a genus was monospecific, we instead fitted an intercept-only model without the species-level random intercept term. For climatic centers, we selected mean annual temperature (BIO1) and annual precipitation (BIO12) as response variables, representing the central tendencies of genus-level climatic distributions. For thermal limits, we selected mean daily maximum temperature of the warmest month (BIO5) and mean daily minimum temperature of the coldest month (BIO6) to capture the upper and lower bounds of genus-level thermal tolerances. Although we treat responses of both climatic centers and thermal limits as multivariate in the modeling framework for convenience, mathematically they are modeled independently without correlation of residuals.

For a given genus, with species i and observations j, the temperature response (i.e., BIO1, BIO5, and BIO6) at each occurrence point, denoted ${y}_{{ij}}$, was modeled using a normal distribution conditional on a species-level random intercept ${b}_{i}$ with phylogenetic variance-covariance matrix ${{{\bf{A}}}}$ $\left(1\right)-(3)$:

1 $$\left.{y}_{{ij}}\right|{b}_{i} \sim {{{\mathscr{N}}}}\left({\mu }_{i},{\sigma }_{\epsilon }^{2}\right)$$

2 $${\mu }_{i}=\beta+{b}_{i}$$

3 $${b}_{i} \sim {{{\mathscr{N}}}}\left(0,{\sigma }_{b}^{2}{{{\bf{A}}}}\right)$$

where ${\sigma }_{b}^{2}$ and ${\sigma }_{\epsilon }^{2}$ are the between-species and within-species variances, respectively, and β is the genus-level fixed intercept. For annual precipitation, since it is continuous and non-negative, we instead used a Gamma distribution with the mean ${\mu }_{i}$ and shape α parameterization $\left(4\right)-(6)$:

4 $$\left.{y}_{{ij}}\right|{b}_{i} \sim {{{\rm{Gamma}}}}\left({\mu }_{i},\alpha \right)$$

5 $${\mu }_{i}=\beta+{b}_{i}$$

6 $${b}_{i} \sim {{{\mathscr{N}}}}\left(0,{\sigma }_{b}^{2}{{{\bf{A}}}}\right)$$

For each genus, we aimed to estimate the fixed intercepts β, which represented our average genus-level climatic centers and thermal limits. We fitted the models under a Bayesian framework using the “brms” R package^85^. We used Markov chain Monte Carlo sampling with four chains of 2000 iterations each (1000 warm-up per chain), resulting in 4000 iterations post-warm-up. To improve model convergence, we specified the following broad priors for $\beta \,\left(7\right)-(10)$:

7 $${\beta }_{{BIO}1}{{{\mathscr{ \sim }}}}{{{\mathscr{N}}}}\left(10,{5}^{2}\right)$$

8 $${\beta }_{{BIO}12}{{{\mathscr{ \sim }}}}{{{\mathscr{N}}}}\left(1000,\,{500}^{2}\right),{{{\rm{lower\; bound}}}}=0$$

9 $${\beta }_{{BIO}5}{{{\mathscr{ \sim }}}}{{{\mathscr{N}}}}\left(25,{5}^{2}\right)$$

10 $${\beta }_{{BIO}6}{{{\mathscr{ \sim }}}}{{{\mathscr{N}}}}\left(5,{5}^{2}\right)$$

For all other parameters, we used the default weakly informative priors. The likelihood contribution of each occurrence point was weighted as described in the previous section. We set the maximum tree depth to 12 and adapted delta (the target average acceptance probability of the sampler) to 0.99 for all models. Final model convergence was assessed using the Gelman-Rubin criterion ($\hat{R}$). Most models were found to converge without any issues ($\hat{R}\approx 1.0$). For climatic centers, we excluded any models with $\hat{R} > 1.05$ or substantial divergent transitions ( > 40) from plots. But for thermal limits, we instead labeled models that exceeded common $\hat{R}$ thresholds (*: $\hat{R} > 1.01$ or **: $\hat{R} > 1.05$; all models had <40 divergent transitions).

### Reporting summary

Further information on research design is available in the Nature Portfolio Reporting Summary linked to this article.

## Supplementary information

Supplementary Information

Reporting Summary

Transparent Peer Review file

## Data Availability

All data necessary to reproduce our results are freely available either on Zenodo (https://doi.org/10.5281/zenodo.18896284) or can be downloaded from their original sources online. See “data_references.docx” in the Zenodo repository for detailed information on all data sources. The underlying fossil dataset can also be downloaded at https://vfwilkens.github.io/PaleoTrees-WCE/#data.

## Code availability

All code used in this study is available on GitHub (https://github.com/vfwilkens/PaleoTrees-WCE/) and Zenodo (https://doi.org/10.5281/zenodo.18896284).
