# Supplementary material for: Cenozoic losses of tree diversity constrain climatic adaptation of Western and Central European forests
Source: Nat Commun. 2026 Sep 16;17:9879. doi: 10.1038/s41467-026-77806-4 (PMC13582922; doi:10.1038/s41467-026-77806-4)
Supplement: Supplementary file 1 [file 41467_2026_77806_MOESM1_ESM.pdf]

# Cenozoic losses of tree diversity constrain climatic adaptation of Western and Central European forests

## Supplementary Information

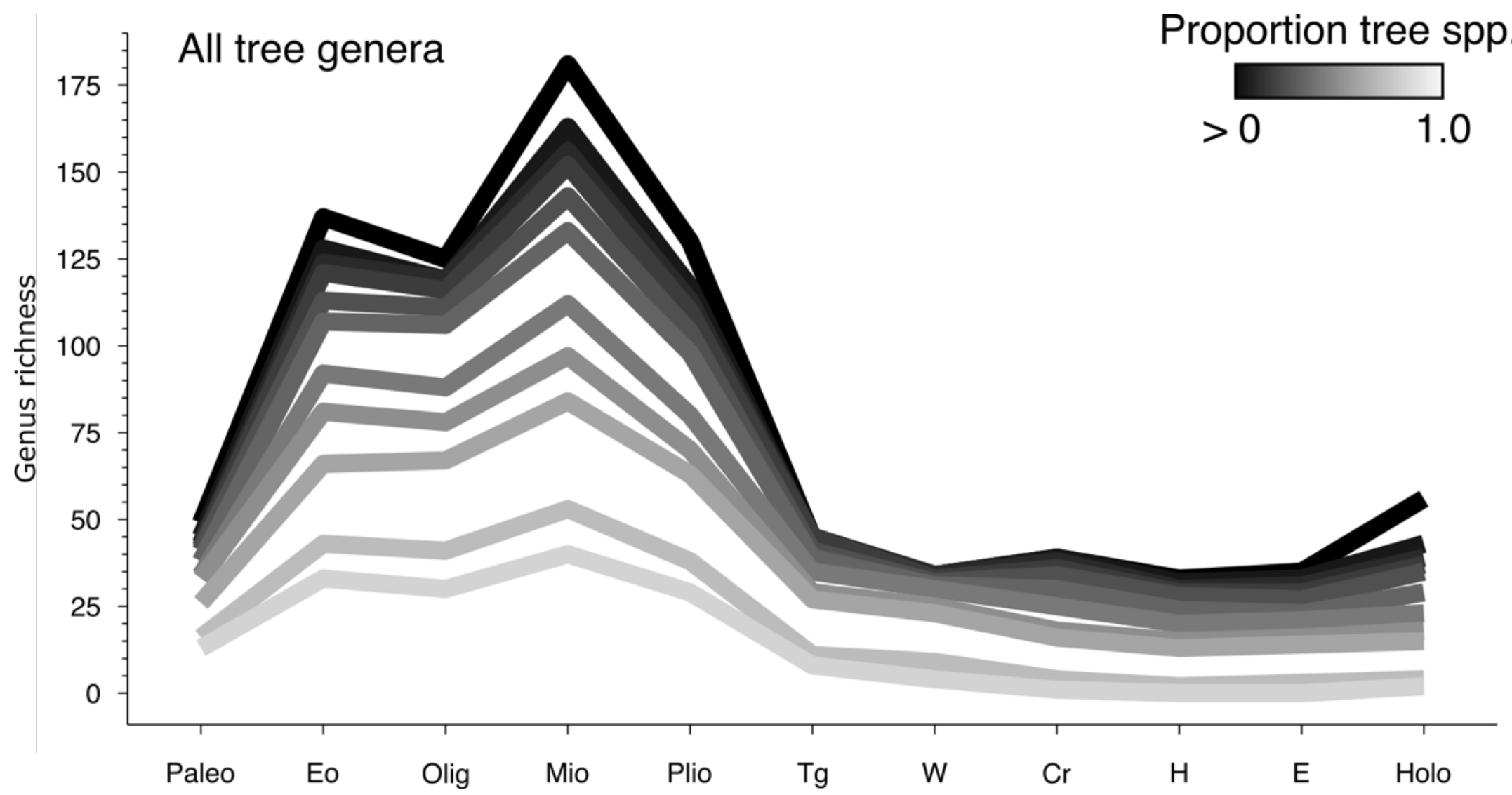


**Supplementary Fig. 1: Changes in tree genus richness during the Cenozoic depending on how a tree genus is defined.** We calculated the proportion of tree species (according to BGCI GlobalTreeSearch database)[1] within each genus (from all accepted species in WCVP)[2] and performed a sensitivity analysis around this value. Only genera containing at least a single tree-forming species were included. Time bin abbreviations are as follows: Paleo – Paleocene, Eo – Eocene, Oligo – Oligocene, Mio – Miocene, Plio – Pliocene, Tg – Tiglian, W – Waalian, Cr – Cromer, H – Holstein interglacial, and E – Eem interglacial, and Holo – Holocene.

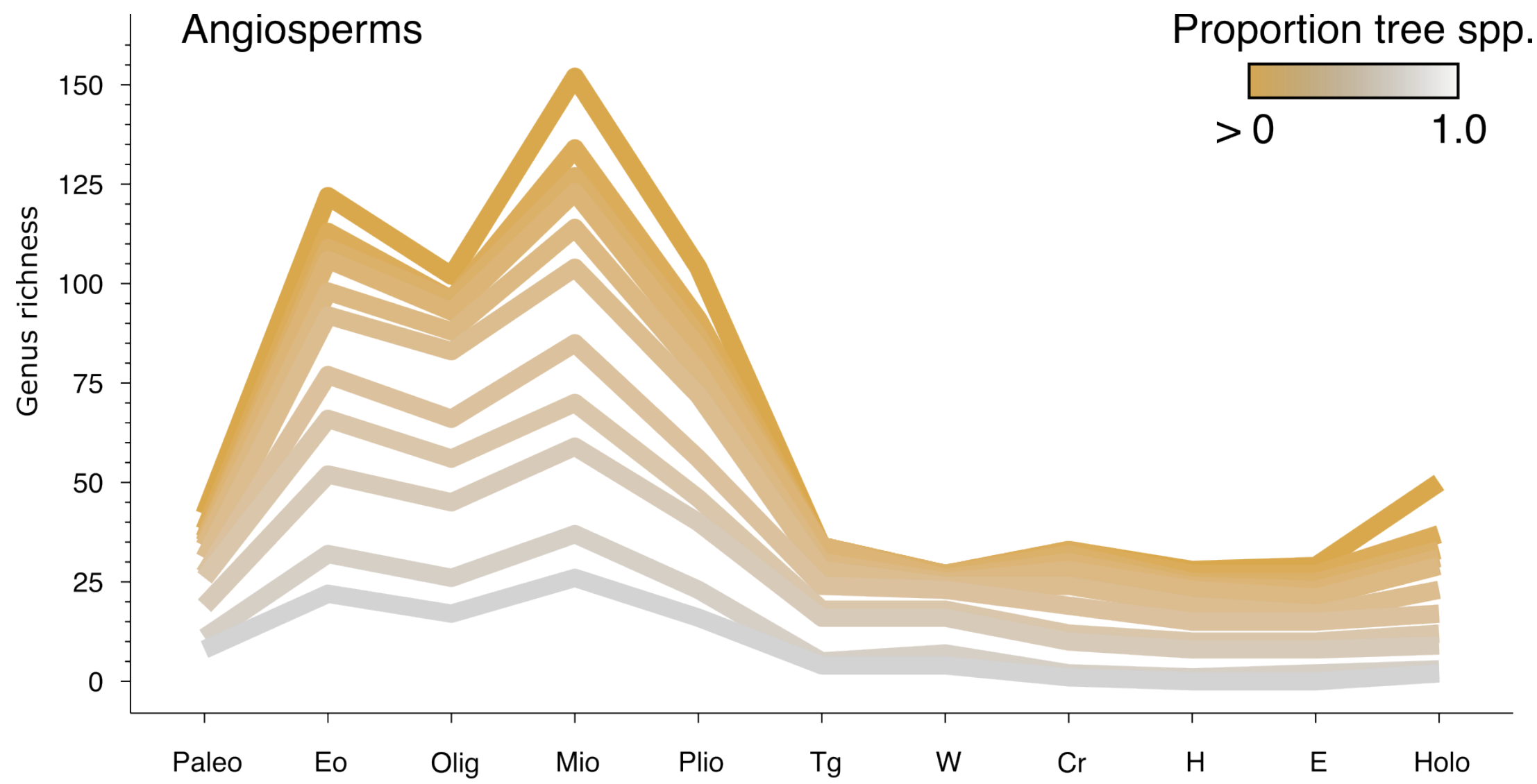


**Supplementary Fig. 2: Changes in tree genus richness of angiosperms during the Cenozoic depending on how a tree genus is defined.** We calculated the proportion of tree species (according to BGCI GlobalTreeSearch database)[1] within each genus (from all accepted species in WCVP)[2] and performed a sensitivity analysis around this value. Only genera containing at least a single tree-forming species were included. Time bin abbreviations are as follows: Paleo – Paleocene, Eo – Eocene, Oligo – Oligocene, Mio – Miocene, Plio – Pliocene, Tg – Tiglian, W – Waalian, Cr – Cromer, H – Holstein interglacial, and E – Eem interglacial, and Holo – Holocene.

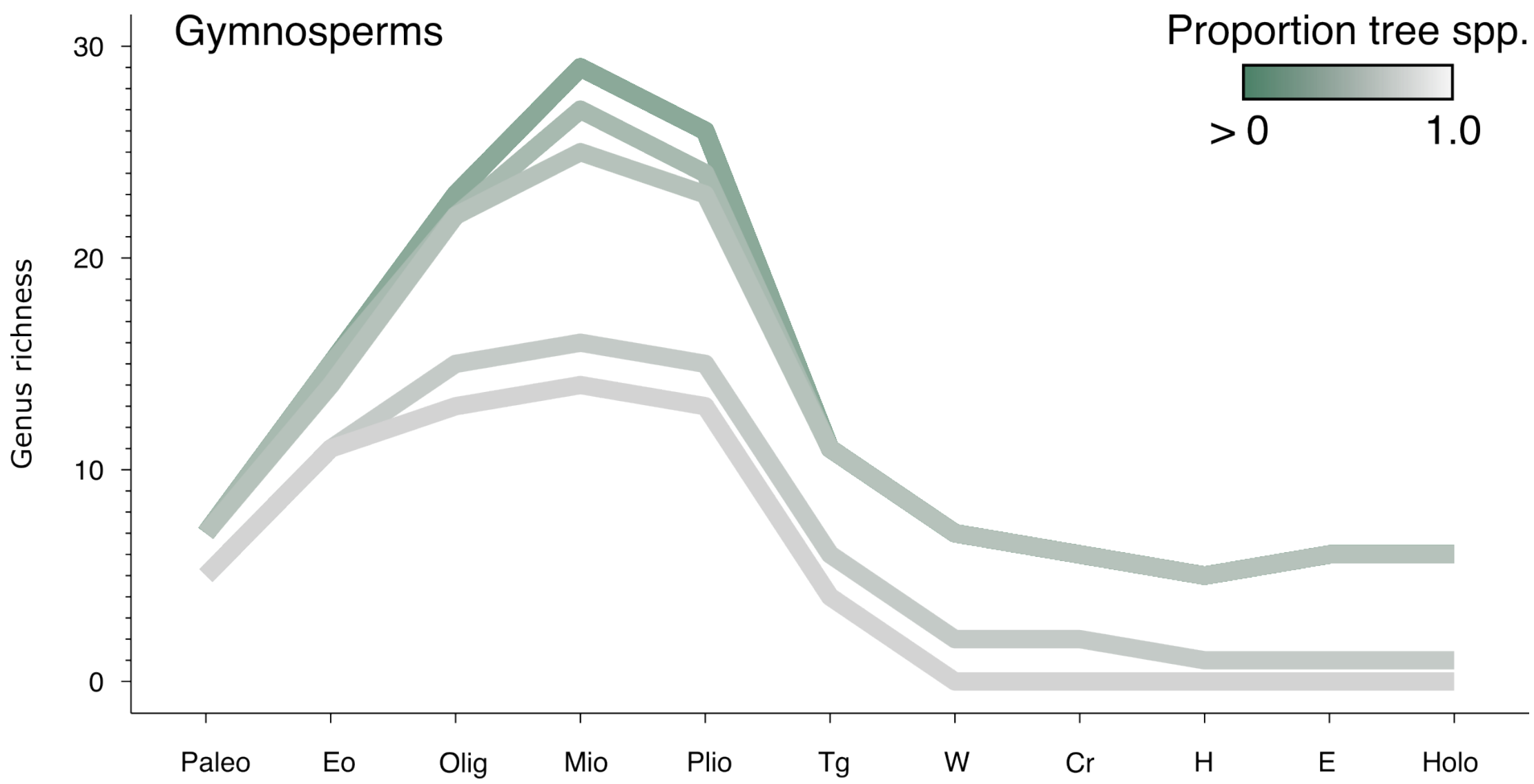


**Supplementary Fig. 3: Changes in tree genus richness of gymnosperms during the Cenozoic depending on how a tree genus is defined.** We calculated the proportion of tree species (according to BGCI GlobalTreeSearch database)[1] within each genus (from all accepted species in WCVP)[2] and performed a sensitivity analysis around this value. Only genera containing at least a single tree-forming species were included. Time bin abbreviations are as follows: Paleo – Paleocene, Eo – Eocene, Oligo – Oligocene, Mio – Miocene, Plio – Pliocene, Tg – Tiglian, W – Waalian, Cr – Cromer, H – Holstein interglacial, and E – Eem interglacial, and Holo – Holocene.

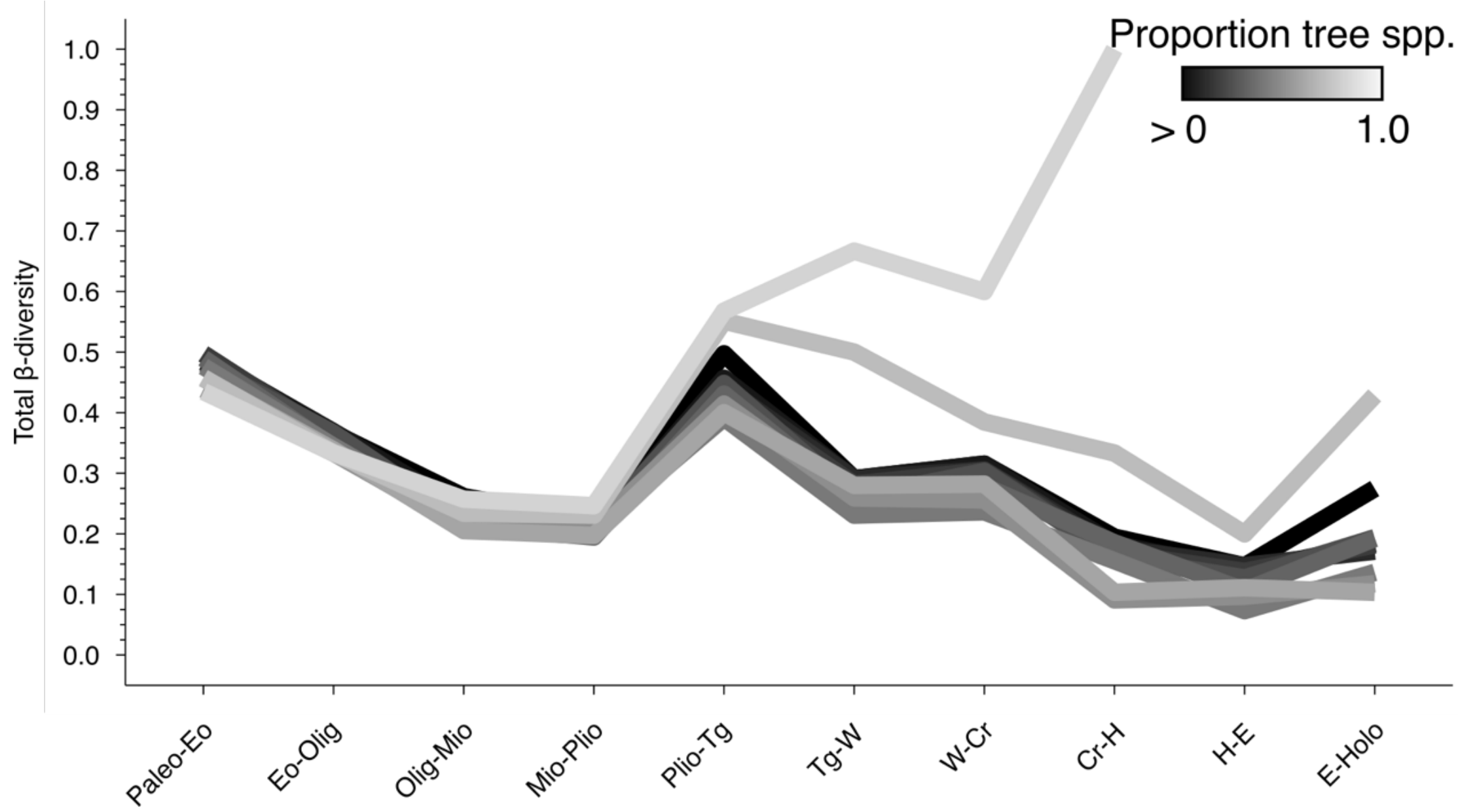


**Supplementary Fig. 4: Changes in total beta diversity between subsequent time bins during the Cenozoic, depending on how a tree genus is defined.** We calculated the proportion of tree species (according to BGCI GlobalTreeSearch database)[1] within each genus (from all accepted species in WCVP)[2] and performed a sensitivity analysis around this value. Only genera containing at least a single tree-forming species were included. Time bin abbreviations are as follows: Paleo – Paleocene, Eo – Eocene, Oligo – Oligocene, Mio – Miocene, Plio – Pliocene, Tg – Tiglian, W – Waalian, Cr – Cromer, H – Holstein interglacial, and E – Eem interglacial, and Holo – Holocene.

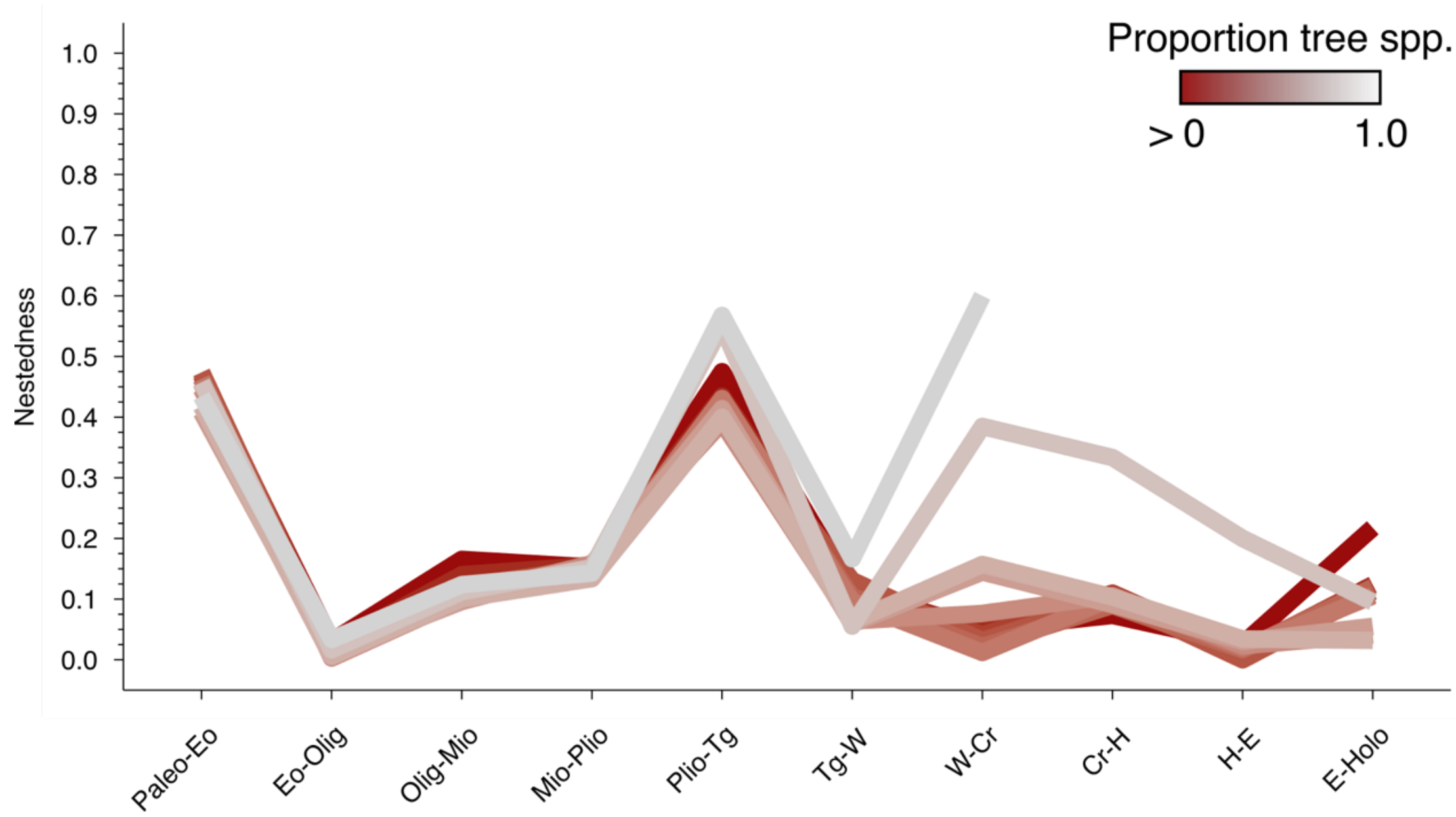


**Supplementary Fig. 5: Changes in the nestedness component of total beta diversity between subsequent time bins during the Cenozoic, depending on how a tree genus is defined.** We calculated the proportion of tree species (according to BGCI GlobalTreeSearch database)[1] within each genus (from all accepted species in WCVP)[2] and performed a sensitivity analysis around this value. Only genera containing at least a single tree-forming species were included. Time bin abbreviations are as follows: Paleo – Paleocene, Eo – Eocene, Oligo – Oligocene, Mio – Miocene, Plio – Pliocene, Tg – Tiglian, W – Waalian, Cr – Cromer, H – Holstein interglacial, and E – Eem interglacial, and Holo – Holocene.

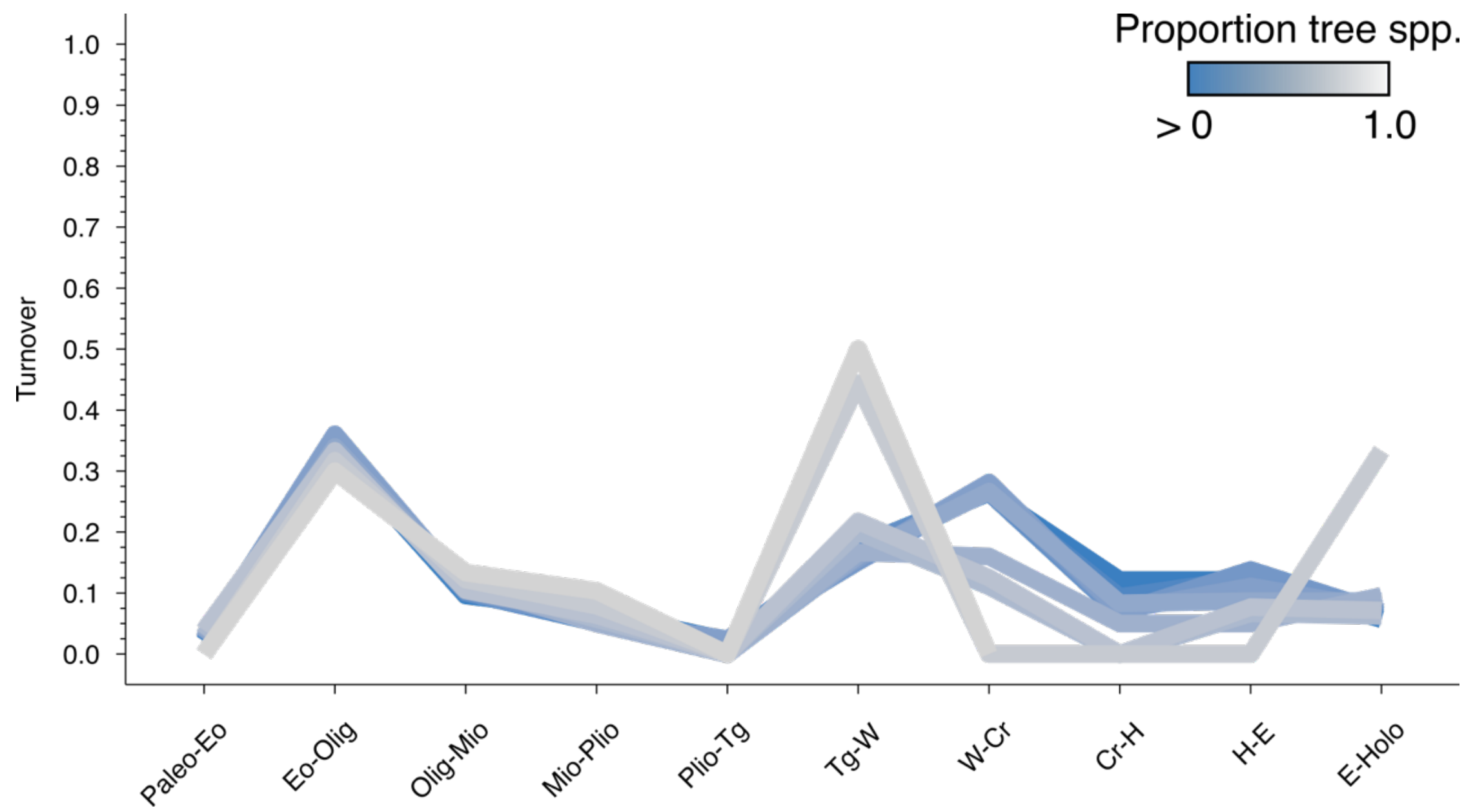


**Supplementary Fig. 6: Changes in the turnover component of total beta diversity between subsequent time bins during the Cenozoic, depending on how a tree genus is defined.** We calculated the proportion of tree species (according to BGCI GlobalTreeSearch database)[1] within each genus (from all accepted species in WCVP)[2] and performed a sensitivity analysis around this value. Only genera containing at least a single tree-forming species were included. Time bin abbreviations are as follows: Paleo – Paleocene, Eo – Eocene, Oligo – Oligocene, Mio – Miocene, Plio – Pliocene, Tg – Tiglian, W – Waalian, Cr – Cromer, H – Holstein interglacial, and E – Eem interglacial, and Holo – Holocene.

**Supplementary Fig. 7**

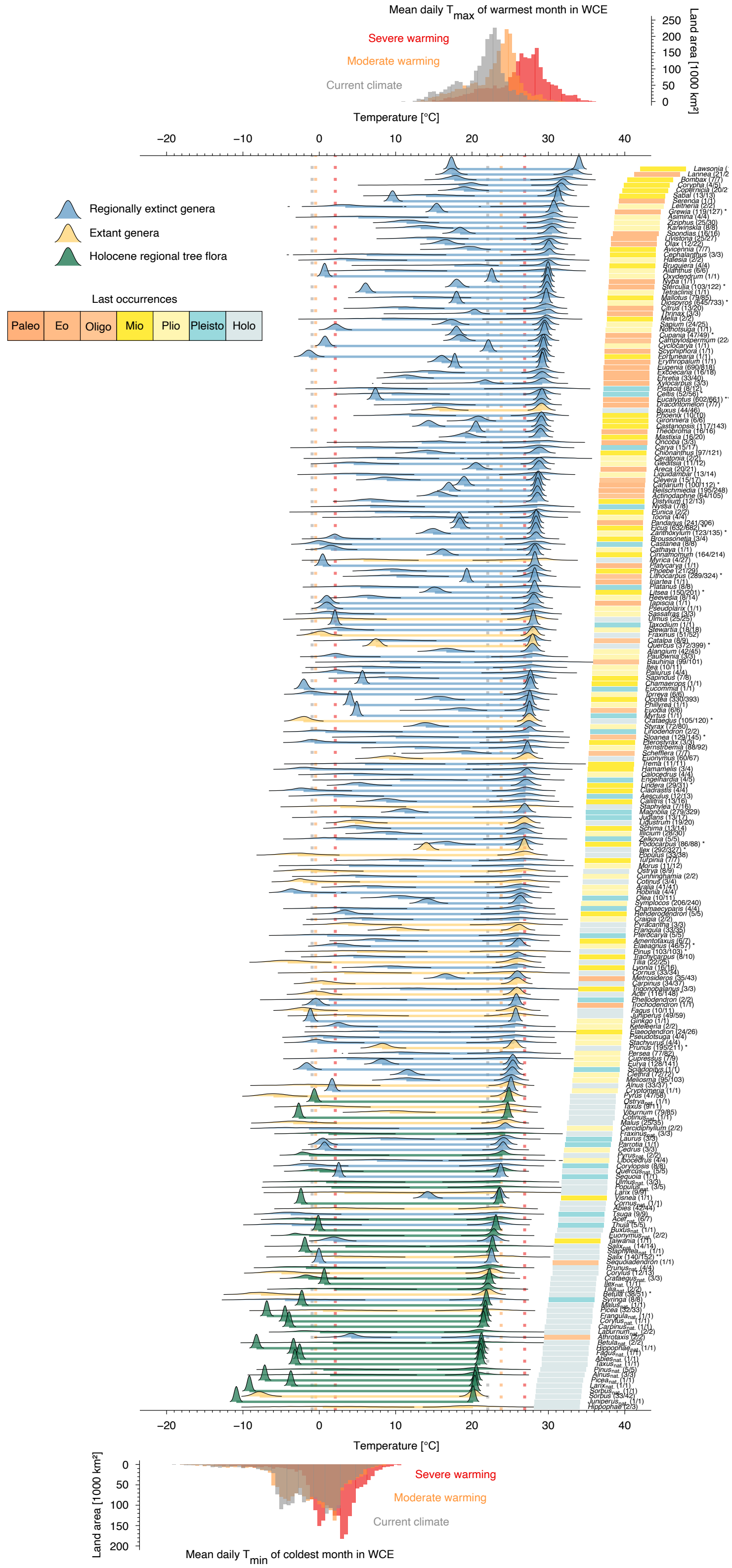

**Supplementary Fig. 7: Upper and lower thermal limits of Cenozoic tree genera from Western and Central Europe.** Posteriors of upper and lower thermal limits for 200 tree genera were estimated, comparing (i) regionally extinct genera (blue), (ii) extant genera (yellow), and (iii) the current option-space for forest reorganization, referred to as the Holocene regional tree flora (green). Thermal limits of regionally extinct and extant genera were estimated using all available tree species within each genus globally, whereas thermal limits of the Holocene regional tree flora were estimated for each extant genus using only the native species pools of Western and Central Europe. Models were arranged by increasing upper thermal limit (bottom to top). Color stripes correspond to the last known occurrence of each genus in the fossil record of Western and Central Europe. All genera have been labelled on the right-hand side of the plot, including the number of species from all accepted species in each genus used to fit the models. The Gelman-Rubin criterion ($\hat{R}$) was used to assess Bayesian model convergence, with values closer to 1.0 indicating successful convergence. Genera with $\hat{R}$ > 1.01 (*) or $\hat{R}$ > 1.05 (**) were labelled as having potentially unreliable estimates. The distribution of heat extremes (mean daily maximum temperature of the warmest month) and cold extremes (mean daily minimum temperature of the coldest month) by land area under current and future climate scenarios in Western and Central Europe are overlaid[3,4]. Current climate: 1981 – 2010. Moderate warming: SSP1-2.6 (+ 1.3 – 2.4 °C by 2100). Severe warming: SSP5-8.5 (+ 3.3 – 5.7 °C by 2100).

## Supplementary References


1. B.G.C.I. GlobalTreeSearch online database. Botanic Gardens Conservation International. (2024).
2. Govaerts, R., Nic Lughadha, E., Black, N., Turner, R. & Paton, A. The World Checklist of Vascular Plants, a continuously updated resource for exploring global plant diversity. *Sci Data* **8**, 215 (2021).
3. Karger, D. N. *et al.* Climatologies at high resolution for the earth's land surface areas. *Sci Data* **4**, 170122 (2017).
4. Brun, P., Zimmermann, N. E., Hari, C., Pellissier, L. & Karger, D. N. Global climate-related predictors at kilometer resolution for the past and future. *Earth System Science Data* **14**, 5573–5603 (2022).
