# Supplementary material for: Cenozoic losses of tree diversity constrain climatic adaptation of Western and Central European forests
Source: Nat Commun. 2026 Sep 16;17:9879. doi: 10.1038/s41467-026-77806-4 (PMC13582922; doi:10.1038/s41467-026-77806-4)
Supplement: Supplementary file 3 [file 41467_2026_77806_MOESM3_ESM.pdf]

nature portfolio

Peer Review File

Cenozoic losses of tree diversity constrain climatic adaptation of Western and Central European forests

Corresponding Author: Mr Vincent Wilkens

This file contains all reviewer reports in order by version, followed by all author rebuttals in order by version.

Version 0:

Reviewer comments:

Reviewer #1

(Remarks to the Author)
By compiling a data set of published/fossil occurrences of tree genera (i.e. genera with above 30% of their species that are arboreal) in Western and Central Europe, the authors analyse its change along the Cenozoic. The manuscript includes a niche model analyses to explore climate changes by comparing extant and extinct species. The manuscript is of high interest providing a good revision of the biodiversity evolution of European tree genera, and giving light to forest managers to consider certain genera to guide assisted forest adaptation to future scenarios.

Below, I detail several key concerns that, from a scientific perspective, must be resolved to ensure the robustness and validity of the findings presented in this manuscript:

“The claim that "a complete overview of tree diversity during the Cenozoic is lacking" (line 46), requires qualification and proper citation. For Western and Central Europe, in particular, several foundational reviews offer comprehensive overviews of Cenozoic tree diversity, including those by Mai (various publications) and Collinson, Culver, & Rawson (2000) (Cenozoic evolution of modern plant communities and vegetation. Biotic responses to global change: the last 145 million years, 223-243), among others. These established works should be referenced and discussed to provide a complete background.
Regarding lines 253–254, the statement that "previous studies... were conducted at a much coarser temporal resolution" seems to dismiss established research without justification. This claim is problematic because it omits a necessary review of regional reference works that specifically detail Cenozoic (or period-specific) floristic changes in the study area.
Line 371. Pleistocene and Holocene fossil occurrences were excluded from the search in the Palaeobiology database. Why using this database (cf. EDP/Neotoma) Explain the reason to exclude Pleistocene from data bases.
Line 67. Clarification is needed on the definition and verification of "precisely dated" records. How was the precision checked? The authors should explain their criteria for accepting the temporal data, detailing whether they checked the validity of age-depth models
Lines 386-394. (1) The authors must clarify their treatment of Holocene fossil occurrences. It appears they are considering them as modern presence data (Lines 392-393) rather than as part of the fossil record. Clarification is needed on how these Holocene occurrences were obtained and whether their temporal resolution was accounted for in the analysis, as treating occurrences spanning 11,700 years as synchronous modern data could lead to methodological errors.
Exclusion of genera native only to France led to the consideration of genera such as Laurus, Pistacia or Celtis, but may be Juglans and Castanea too, as non-native in Central/Western Europe. This exclusion criterion should be carefully reassessed (like e.g Carrión, J. S., & Sánchez-Gómez, P. 1992). In the context of restoring lost tree diversity (as discussed in line 301), I am concerned about labelling heat-tolerant species that are native to southern France as non-native for the broader Central/Western European region (see line 29). There is a specific pool of Mediterranean species present in Southern/Western Europe that warrants a different consideration in diversity restoration efforts. It also applies to temperate species like Juglans regia or Castanea sativa (cf. line 315), already present in Central-Western Europe (see gbif.org). These taxa should be distinguished from genuinely non-native, potentially invasive species (line 303) that threaten native biodiversity. The warn given in lines 299-301 should be qualify in light of species already present in the Western-Present Europe (checking the non-native consideration in this paper)
Niche models: The manuscript currently lacks citations for the reference works that guided the niche modeling analysis. More critically, the authors must explicitly justify the choice of using Phylogenetic Generalized Linear Mixed Models. This approach should be compared against other alternatives (e.g., standard GLMs, Maximum Entropy Models, ensemble models, etc.) to clearly articulate the benefits gained.

The authors should describe the validation method used for the models. Typically, this involves a procedure like data partitioning (e.g., k-fold cross-validation or training/testing splits), and the metrics used to assess model performance. Finally, there is a discrepancy in data resolution that requires explanation. The manuscript states that the occurrence information has an accuracy of <10 km (line 410) and the original climatic information is at 1 km resolution (line 416). The authors then state they aggregated the climatic data to 25 km (line 418). The rationale for choosing to aggregate the high-resolution climatic data to a much coarser resolution, thereby losing informative variance, must be justified.

(Remarks on code availability)

Reviewer #2

(Remarks to the Author)
The authors tried to use the fossil dataset of tree genera from Western and Central Europe to analyze tree diversity changes during the Cenozoic. This is a very interesting and useful work, especially for their implications for the adaptation of the region's ecosystems to ongoing climate change. The authors given the dynamic change trend of tree genus richness, and most of the tree genus losses are related to heat-tolerant lineages. Finally, this work provided several ways that inform forest management to increase regional tree diversity and enhance forest resilience under climate change based on paleoecological data.
I have several concerns for this work.
First, I see the authors compiled a dataset of macrofossil and fossil pollen occurrences of tree genera. But I haven't seen where is the dataset. This is very important for this work.
Second, the analysis only focus on the tree genera in Western and Central Europe, why not include Northern Europe and Southern Europe in the analysis?
Third, many genera have very large latitude span, while the species occurrence data was downloaded from GBIF for all tree species belonging to genera in the final fossil occurrence dataset. Their climate niche evolution maybe change to diversity along their dispersal and new habitat. Thus, the reasonability to use the current climate data of all species in the genera is open to discussion.

(Remarks on code availability)

Reviewer #3

(Remarks to the Author)
The paper is quite innovative: it presents a complex processing of Cenozoic record of 200 modern tree genera in order to depict some climate changes in past as well as to give some predictions for forest management within the warming trends in future. I consider it as fully acceptable for being published in Nature Communications !

I put all my comments / questions or even corrections directly in the annotated version of the manuscript, in the attached file called "652672_0_art_file_294013_t2bym8-js.pdf", so only general points :

I must openly admit that the statistical part concerning the correct application of R statistical software, concept of PGLMMs and related mathematical issues are rather deep to me, and this part, which very important, must surely by review by someday (skilled statistician), who is familiar with those problems in order to check manuscript independently !

On the other hand, I could give some (I hope constructive) comments relative to the palaeobotanical frame of the manuscript. Here, I think as very preliminary and also most important step is the correct choice of 200 modern genera and their distribution in time and space (WCE). As I noticed in my detailed review, I consider PBDB database in its present state as very incomplete so useless… In other words, today, its application can be very misleading – in this respect, the most important part of the manuscript to me as a palaeobotanist is about a correct application of the references present in the columns "Notes" and "References" in the file called "literature_records_28_01_25.lsx" in Supplementary material - unfortunately, many important pieces of information is relatively hidden there… Generally, there are taxa, which are rather general and cumulative as Ficus (can be related to other types), or Laurus (can you distinguish it from Laurophyllum or other members of Lauraceae, and what about Laurinoxylon and the fossil woods in general?) or others, which can be similar based on foliage to modern ones (e.g., Cercidiphylum, Trigonobalanus), but they in fact represent extinct types as extinct Cercidiphyllum-like plants in Paleocene/Eocene or extinct Trigonobalanopsis/Eotrigonobalanus. Generally, many Paleocene sites in WCE were not systematically revised from "classical" times and there are surely many extinct genera (or stem group member?) as well as in Eocene sites, which are however better known. To sum up, I would put once again a special care to the table "literature_records_28_01_25.lsx" as this is the point of departure for any subsequent analysis and correct results !

More details can be found in the attached files as commentaries, I also add two examples of comparison between PBDB and the "real" fossil record in order to demonstrate incompleteness of that database. However, I am really happy to see such an innovative and complex paper dealing with Central European Cenozoic Palaeobotany !

Jakub Sakala, Kralupy, December 4th, 2025

(Remarks on code availability)
for more details, please, see the two attached files :
1) 652672_0_art_file_294013_t2bym8-js.pdf
2) comparison-record-pbdb-vs-true.rar

Version 1:

Reviewer comments:

Reviewer #1

(Remarks to the Author)
The authors have revised the manuscript and answered the questions raised after first revision, which is highly appreciated, resulting in an improved version of their work. Although the authors have resolved all questions and concerns by amplifying the methodology and discussion sections, there are still a few considerations still worthy to highlight.

1.Regarding the choice of PBDB:

The authors state in their reply that: “In general, fossil pollen records require very careful quality control due to common taphonomic issues, such as older grains being deposited into younger sediments leading to anachronistic “false occurrences” of some taxa. […]. Therefore, it did not make sense to query Neotoma/EDP directly because we assumed that any relevant studies included in these databases would probably appear anyway during literature searches”.
Also they add that “while Neotoma does also contain plant macrofossil occurrences, we checked these and concluded that these occurrences were either redundant or had already been found during our literature search. Thus, they did not add any new information to our dataset”.

First, there appears to be a discrepancy between the authors' response and the text; while the former implies the study relies solely on macrofossils, lines 338–339 indicate the inclusion of fossil pollen. Aside from this specific mention, there are no other references to pollen or macro-remain records throughout the manuscript.

Second, based on the authors’ response, it appears that pollen records were excluded to avoid anachronistic 'false occurrences' arising from taphonomic issues such as redeposition. However, this stance constitutes a significant critique of a vast body of palynological research spanning the Quaternary and Tertiary. It is worth noting that redeposited grains are often distinguishable from contemporaneous pollen by their state of preservation. While pollen data has inherent limitations, it remains a fundamental source of evidence for reconstructing European paleo-flora.

I recommend clarifying if the analysis excludes pollen records. If so, please use the term 'macrofossil' instead of the more general 'fossil' in the appropriate sections (for instance, lines 22, 337, and 364) to more accurately reflect the data source.

2. Regarding the choice of exclusion of Pleistocene occurrences from PBDB:
Authors answer to the raised question: "While PBDB is a great database for deep time occurrences of plant macrofossils, we opted to exclude Pleistocene (and Holocene) occurrences from our query due to issues with chronological precision. In our study, we represent the Pleistocene as five separate time bins based on the chronology from Lang (1994). However, the coarse epoch-level dating in PBDB (e.g., “Pleistocene” or “Late Pleistocene”) was not precise enough to be able to reliably assign occurrences to a specific interglacial period or temperate phase. Therefore, we decided to compile the Pleistocene portion of our dataset exclusively from literature and not from PBDB".

In light of this response, the previously raised question regarding the exclusion of databases such as Neotoma or the European Pollen Database (EPD) becomes even more pertinent. These sources provide a vast number of Quaternary records with greater chronological precision than those typically found in Tertiary studies. If pollen data were indeed utilized, I strongly suggest including a table of the sites and their corresponding references as supplementary material.

3.The treatment of Holocene fossil occurrences:
I previously expressed concern regarding the treatment of Holocene fossil occurrences as modern presence data rather than as part of the fossil record. The authors have clarified that they did not use any Holocene fossil occurrence data in their study, noting that such findings are rare and do not fully represent current native tree diversity in Europe. However, it is widely recognized that Holocene fossil records are significantly more abundant than those from the Pleistocene or Tertiary.

The authors haver also answered that they utilized the World Checklist of Vascular Plants (WCVP) to establish a country-level checklist of native tree genera for Western and Central Europe. First, I have not found any published basis for this specific methodology and suggest adding the appropriate reference. Finally, I also suggest replacing the label 'Holocene' with a more precise term, such as 'W-C Europe Native Status,' to better reflect the nature of the data.

(Remarks on code availability)

Reviewer #2

(Remarks to the Author)
The authors have improved the manuscript very well based on the three reviewers suggestions. I have no other questions for this paper.

(Remarks on code availability)

Reviewer #3

(Remarks to the Author)
Dear Authors,
Congratulations once again on your innovative paper!
And thank you for taking all my comments into account in such detail; this is not common, and I appreciate it all the more.
I fully agree with your revisions and recommend publication in this form.
With warm regards,
Jakub Sakala
P.S.: Perhaps I would just put “form genera” in quotation marks, for example here:
Form genera and other globally extinct taxa (e.g., Laurophyllum)...
since these are now obsolete categories according to the ICBN; only “fossil taxa” is used exclusively, as nicely summarized here:
https://en.wikipedia.org/wiki/Form_classification#In_botany
However, I think you can leave it with the quotation marks, the people will understand :)

(Remarks on code availability)

Open Access This Peer Review File is licensed under a Creative Commons Attribution 4.0 International License, which permits use, sharing, adaptation, distribution and reproduction in any medium or format, as long as you give appropriate credit to the original author(s) and the source, provide a link to the Creative Commons license, and indicate if changes were made.
In cases where reviewers are anonymous, credit should be given to 'Anonymous Referee' and the source.
The images or other third party material in this Peer Review File are included in the article’s Creative Commons license, unless indicated otherwise in a credit line to the material. If material is not included in the article’s Creative Commons license and your intended use is not permitted by statutory regulation or exceeds the permitted use, you will need to obtain permission directly from the copyright holder.
To view a copy of this license, visit https://creativecommons.org/licenses/by/4.0/

REVIEWER COMMENTS

Reviewer #1 (Remarks to the Author):

By compiling a data set of published/fossil occurrences of tree genera (i.e. genera with above 30% of their species that are arboreal) in Western and Central Europe, the authors analyse its change along the Cenozoic. The manuscript includes a niche model analyses to explore climate changes by comparing extant and extinct species. The manuscript is of high interest providing a good revision of the biodiversity evolution of European tree genera, and giving light to forest managers to consider certain genera to guide assisted forest adaptation to future scenarios. Below, I detail several key concerns that, from a scientific perspective, must be resolved to ensure the robustness and validity of the findings presented in this manuscript:

We thank Reviewer #1 for the very thorough and constructive review, which has helped to substantially improve our manuscript!

“The claim that "a complete overview of tree diversity during the Cenozoic is lacking" (line 46), requires qualification and proper citation. For Western and Central Europe, in particular, several foundational reviews offer comprehensive overviews of Cenozoic tree diversity, including those by Mai (various publications) and Collinson, Culver, & Rawson (2000) (Cenozoic evolution of modern plant communities and vegetation. Biotic responses to global change: the last 145 million years, 223-243), among others. These established works should be referenced and discussed to provide a complete background.

This is certainly a fair criticism. While we reference many of these established works throughout (e.g., from Mai, Latham & Ricklefs, Svenning, etc.), we agree that our research gap statement should be put directly into context with these previous works. Therefore, we changed this sentence to emphasize that while several foundational reviews already exist, a modern synthesis that combines these works (in addition to research of the last 20-25 years) and directly links past changes in tree diversity to current challenges in forest reorganization is needed. Furthermore, as you have suggested, we included the reference to Collinson, Culver, & Rawson (2000) and added an additional citation of Mai’s 1994 work to this section.

> REVISED:
> “While several foundational reviews documenting changes in Cenozoic tree diversity exist (e.g., Mai, 1994; Collinson et al., 2000), a modern synthesis linking which taxa were lost, the timing of these losses, and their implications for the adaptation of Western and Central European forests to future climate is still missing.”

Regarding lines 253–254, the statement that "previous studies... were conducted at a much coarser temporal resolution" seems to dismiss established research without justification. This claim is problematic because it omits a necessary review of regional reference works that specifically detail Cenozoic (or period-specific) floristic changes in the study area.

We understand this concern and thank the reviewer for raising it. It was not our intention to dismiss existing works, especially since these regional works are also the foundation of our dataset and our study. Our aim was to emphasize that our study 1) focuses on Western and Central Europe as a whole and 2) provides a continuous overview with 11 time bins in the Cenozoic era. This contrasts with the cited comparable works: Latham & Ricklefs, 1993 (global scope, 2 time bins); Svenning, 2003 (European scope, but only 2 time bins); Eiserhardt et al., 2015 (global scope, only 2 time bins). We have re-written this section to reflect that our study is not "better" but rather a synthesis of these and other works.

> REVISED:
> "Furthermore, our research synthesizes previous global (e.g., Latham & Ricklefs, 1993; Eiserhardt et al., 2015) and regional (e.g., Mai, 1994; Svenning, 2003) studies to provide a continuous, high-resolution overview of tree diversity changes in Western and Central Europe for the entire Cenozoic. This includes five time bins in the Pleistocene that improve coverage when both paleoclimatic variability and extinctions were at their highest."

Line 371. Pleistocene and Holocene fossil occurrences were excluded from the search in the Palaeobiology database. Why using this database (cf. EDP/Neotoma) Explain the reason to exclude Pleistocene from data bases.

Regarding choice of PBDB:

We chose to use PBDB because it provided a strong foundation for compiling deep time plant macrofossil occurrences, which we then significantly expanded upon using literature searches. On the other hand, we opted not to directly query EDP/Neotoma for the following reasons:

- EDP/Neotoma consist of mainly fossil pollen records from the Quaternary (even then, mostly from < 100 kya). This is simply not a very useful basis for building a dataset across the entire Cenozoic era (66 Ma – present).
- In general, fossil pollen records require very careful quality control due to common taphonomic issues, such as older grains being deposited into younger sediments leading to anachronistic "false occurrences" of some taxa. In our experience, the underlying studies of the fossil pollen records must always be carefully reviewed regarding how authors interpreted certain grains as being, for example, likely redeposits and not reliable evidence of a taxa's presence. Therefore, it did not make sense to query Neotoma/EDP directly because we assumed that any relevant studies included in these databases would probably appear anyway during literature searches.
- Lastly, while Neotoma does also contain plant macrofossil occurrences, we checked these and concluded that these occurrences were either redundant or had already been found during our literature search. Thus, they did not add any new information to our dataset.

We added a sentence after the first mention of PBDB in the Methods briefly justifying its use.

NEW:

"PBDB is a paleontological database that was chosen as the basis for our fossil dataset because it is curated and contains standardized fossil occurrences across all geological ages."

Regarding exclusion of Pleistocene occurrences from PBDB:

While PBDB is a great database for deep time occurrences of plant macrofossils, we opted to exclude Pleistocene (and Holocene) occurrences from our query due to issues with chronological precision. In our study, we represent the Pleistocene as five separate time bins based on the chronology from Lang (1994). However, the coarse epoch-level dating in PBDB (e.g., "Pleistocene" or "Late Pleistocene") was not precise enough to be able to reliably assign occurrences to a specific interglacial period or temperate phase. Therefore, we decided to compile the Pleistocene portion of our dataset exclusively from literature and not from PBDB. Please see also further below the explanation regarding Holocene occurrences.

To clarify these decisions in the manuscript, we have updated the Methods section accordingly.

NEW:

"Since dating of Pleistocene occurrences was too coarse to assign these to a specific interglacial period or temperate phase based on the chronology from Lang (1994), we excluded these from the PBDB dataset. Instead, occurrences for the Pleistocene as well as the Holocene were obtained from other sources (see explanation below)."

Line 67. Clarification is needed on the definition and verification of "precisely dated" records. How was the precision checked? The authors should explain their criteria for accepting the temporal data, detailing whether they checked the validity of age-depth models

Thanks for pointing this out. We did not mean to give the impression that we independently recalculated or checked the validity of age-depth models. We agree that the qualifier "precisely" could be misleading in this context. We have removed this term from the manuscript to prevent any confusion.

REVISED:

"We also identified fossil occurrences dated to five temperate stages and interglacial periods within the Pleistocene, improving the temporal resolution of our dataset in the geologically recent past."

Regarding our criteria for accepting temporal data, we relied entirely on previous verification via data curators (PBDB) and peer-review. For data sourced directly from literature, we assessed each study individually and accepted it only if the biostratigraphy or dating methods were explicitly described in the text as well as review articles. Since our dataset is built largely from peer-reviewed literature (except for a few academic books like Lang, 1994), we relied mainly on the original peer-review process underlying each published source to ensure the general validity of the described dating methods and stratigraphy.
We have added a sentence in the Methods section where we clarify these criteria.

NEW:

"We focused mainly on peer-reviewed literature and accepted a study if the biostratigraphy or dating methods were explicitly described in the text or if it was a review-type article."

Lines 386-394. (1) The authors must clarify their treatment of Holocene fossil occurrences. It appears they are considering them as modern presence data (Lines 392-393) rather than as part of the fossil record. Clarification is needed on how these Holocene occurrences were obtained and whether their temporal resolution was accounted for in the analysis, as treating occurrences spanning 11,700 years as synchronous modern data could lead to methodological errors.

We thank the reviewer for raising this point. However, we would like to clarify that we did not use any Holocene fossil occurrence data in our study. In general, fossil findings from the Holocene are very rare and do not represent the full spectrum of native tree diversity in Europe today. There are only 15 tree genera listed for the Holocene in the data that we queried from PBDB. Major genera like *Tilia* or *Abies* are missing. Instead, we used the World Checklist of Vascular Plants (WCVP) to build a country-level checklist of native tree genera for Western and Central Europe. This approach was considerably less biased than relying on Holocene fossils (or treating Holocene fossils as equal to modern occurrences). This resulted in a checklist of 37 tree genera being identified as native rather than only 15 from PBDB.

We reworded this section of the Methods to avoid further confusion and make it clear we did not use Holocene fossil occurrences in our study.

REVISED:

"Lastly, to determine the presence of genera in Western and Central Europe during the Holocene, we could not use fossil occurrences due to the scarcity of findings. Instead, we obtained a modern checklist of accepted native tree genera for Western and Central Europe using WCVP, accessed via the "rWCVP" R package (Brown et al., 2023)."

Exclusion of genera native only to France led to the consideration of genera such as Laurus, Pistacia or Celtis, but may be Juglans and Castanea too, as non-native in Central/Western Europe. This exclusion criterion should be carefully reassessed (like e.g Carrión, J. S., & Sánchez-Gómez, P. 1992).
In the context of restoring lost tree diversity (as discussed in line 301), I am concerned about labelling heat-tolerant species that are native to southern France as non-native for the broader Central/Western European region (see line 29). There is a specific pool of Mediterranean species present in Southern/Western Europe that warrants a different consideration in diversity restoration efforts.
It also applies to temperate species like Juglans regia or Castanea sativa (cf. line 315), already present in Central-Western Europe (see gbif.org). These taxa should be distinguished from genuinely non-native, potentially invasive species (line 303) that threaten native biodiversity. The warn given in lines 299-301 should be qualify in light of species already present in the Western-Present Europe (checking the non-native consideration in this paper)

Thanks for this comment! There are quite a few interesting points raised here that we will try to address together below.

The tree genera that were considered non-native to Western and Central Europe because of the exclusion of France are *Juglans*, *Celtis*, *Paliurus*, *Laurus*, *Pistacia*, *Myrtus*, *Chamaerops*, *Phillyrea*, and *Ceratonia*. First of all, we added the full list to the Methods to improve transparency about this decision. We also added labels for all nine of these genera to Fig. 4a, so the reader can evaluate this decision themselves.

NEW:

"These genera were *Juglans*, *Celtis*, *Paliurus*, *Laurus*, *Pistacia*, *Myrtus*, *Chamaerops*, *Phillyrea*, and *Ceratonia*."

REVISED:

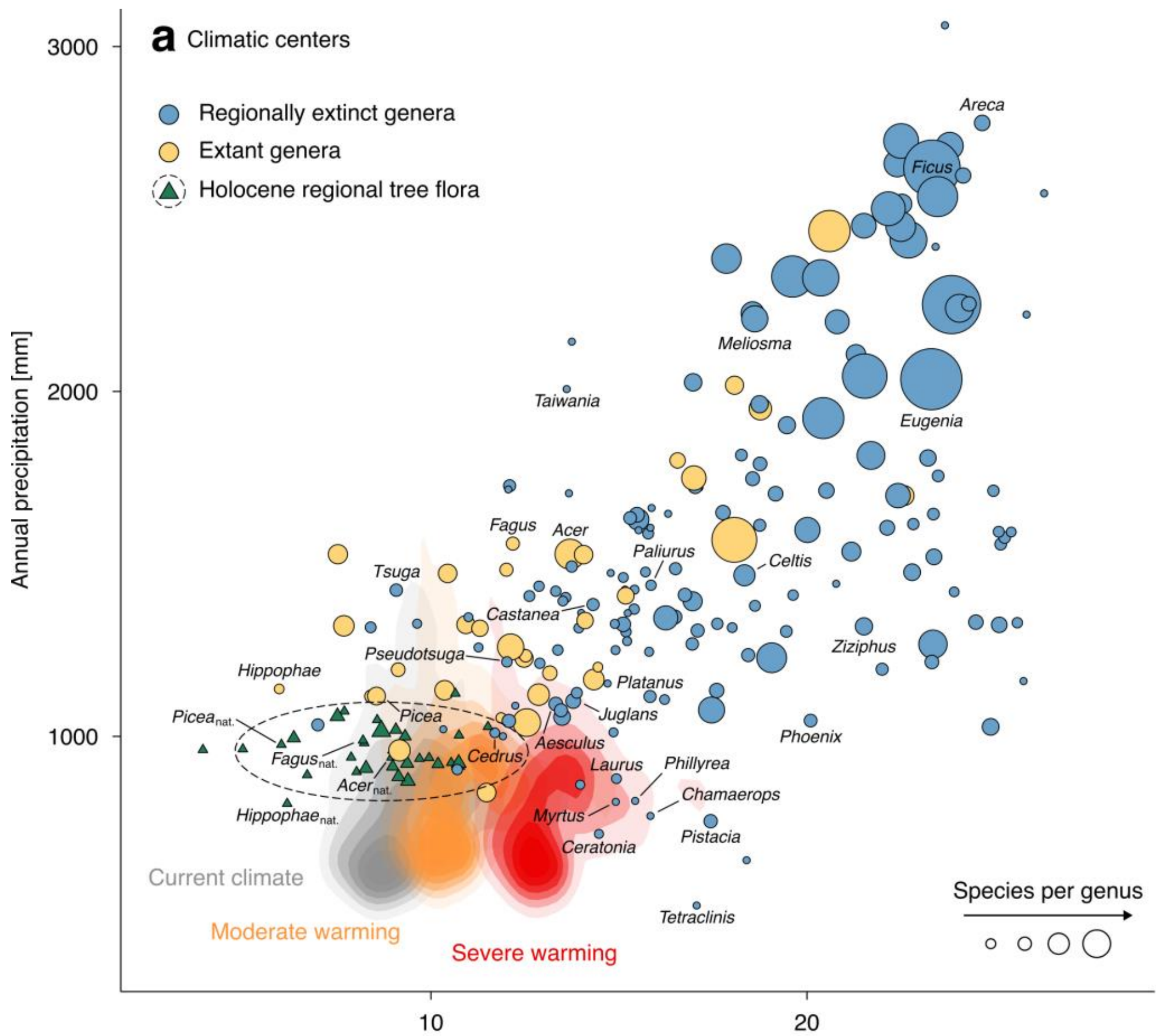

We used country-level checklists from WCVP to create a single checklist of tree genera native to Western and Central Europe. Of course, such checklists are inherently coarse and do not consider large regional floristic differences within countries themselves. This problem was most apparent in France, where subtropical Mediterranean ecosystems contrast with the greater temperate forest zone, the latter of which is the focus of our study.

We thought about some alternative approaches, such as considering these genera as native after all or using more high resolution data. However, we think regardless of the chosen method, there will always be some borderline cases of genera that are either included or excluded, depending on what filters or sources are used to build the checklist. This is a problem that is simply inherent to trying to generate a native tree genus checklist for such a large heterogenous study region. We think the best approach is to be more transparent about this choice and expand the discussion regarding the role of these genera in the future.

We completely agree that the European biogeographical histories of broadleaf deciduous genera from the Mediterranean basin like *Juglans* or *Castanea* are complex and cannot be easily disentangled from human history. Because of their long-standing history of cultivation by humans since thousands of years or more, including in Western and Central Europe, we must distinguish them from other non-native or potentially invasive taxa introduced only very recently. At the same time, there are also thermophilus evergreen genera like *Laurus* or *Pistacia*, which have not played a role in current temperate forest ecosystems during the Holocene, but have a limited presence in Southern France that could increase in the future because of phytoclimatic shifts (Conradi et al., 2024).

In summary, we think the best approach to this concern is to emphasize these nuances in the Discussion. We have revised the end of the Discussion accordingly to highlight the points raised above and added some relevant references, including the suggested study.

> REVISED:
> “In particular, genera that were present in Western and Central Europe during the Pleistocene but persisted into the Holocene only in neighboring regions could be promoted using assisted migrations (e.g., broadleaved-deciduous species from Southern Europe like Juglans regia L. or Castanea sativa Mill.; Kreyling et al., 2011). The European biogeographical histories of some of these genera are still unresolved, as millennia of human cultivation, including in Western and Central Europe, obscure their natural post-glacial distributions (Carrion & Sanchez-Gomez et al., 1992; Pollegioni et al., 2017; Roces-Díaz et al., 2018). Other Mediterranean genera, like Laurus or Pistacia, which we considered non-native to Western and Central Europe in our study, could become more prominent in the future as phytoclimatic shifts increasingly favor evergreen trees over needleleaf and cold-deciduous trees (Conradi et al., 2024). Considering these nuances, tree genera from neighboring regions warrant special consideration under forest reorganization in contrast to non-native genera from other continents like Pseudotsuga, which was introduced into the region only in the last two centuries.”

Niche models:
The manuscript currently lacks citations for the reference works that guided the niche modeling analysis. More critically, the authors must explicitly justify the choice of using Phylogenetic Generalized Linear Mixed Models. This approach should be compared against other alternatives (e.g., standard GLMs, Maximum Entropy Models, ensemble models, etc.) to clearly articulate the benefits gained. The authors should describe the validation method used for the models. Typically, this involves a

procedure like data partitioning (e.g., k-fold cross-validation or training/testing splits), and the metrics used to assess model performance.

Thanks for the suggestions. We have added a comparison with GLMs and GLMMs in the Methods section, explaining the advantages of using PGLMMs to fit genus-level niche models. We have also added references to this part as requested.

> NEW:
> "There are several critical advantages to using PGLMMs to fit genus-level niche models over alternative approaches (Smith et al., 2019). First, we can directly account for both the statistical non-independence of occurrence data within species as well as the phylogenetic non-independence of species within genera (Joly & Wolkovich, 2019). With GLMs, occurrences across species are assumed to be statistically independent, which severely biases the inference of genus-level niches towards species that are overrepresented in the data (e.g., Pinus sylvestris in Pinus). While GLMMs account for statistical non-independence of occurrences, they still assume that species within a genus are statistically independent from one another. PGLMMs overcome these problems by incorporating shared evolutionary history of species through partial pooling, which is a feature of hierarchical models that enables information sharing across grouping variables (Godoy et al., 2018). In PGLMMs, the degree of information sharing is inversely proportional to the phylogenetic distance between species, which is regulated by a phylogenetic variance-covariance matrix. As a result, species-level parameters are adjusted toward genus-level estimates in a way that reflects shared evolutionary history and ecological limits within genera. Furthermore, this approach also improves the reliability of parameter estimates for rare species, compensating for sparse occurrence data by borrowing strength from common species through shared evolutionary relationships (Lomba et al., 2010; Ovaskainen et al., 2025)."

The suggested model validation approaches (e.g., k-fold CV, training/test splits, etc.) or performance metrics like AUC/TSS are designed to test the predictive accuracy of a spatial classifier, i.e., of species distribution models (SDMs). However, our objective is not to predict spatial distributions but to infer genus-level niches. This is strictly a presence-only approach that does not use absence or pseudo-absence data. Therefore, we cannot incorporate the suggested performance metrics or directly compare our niche inference approach to SDMs like MaxEnt or ensemble models, as the underlying goal of each approach is fundamentally different. While GLMs/GLMMs could also be used to infer genus-level niches, we have outlined in the Methods section why their use here would be inappropriate compared to PGLMMs. In summary, GLMs/GLMMs assume that occurrences and species are statistically and phylogenetically independent. However, this assumption does not hold due to several biases in the data, which is why we must use PGLMMs to account for this.

"Validation" within an inferential Bayesian framework consists of assessing model convergence and parameter uncertainty. In our study, we assessed model convergence using the Gelman-Rubin criterion ($\hat{R} < 1.05$ or sometimes more strictly < 1.01, depending on who you ask). We also excluded (in Fig. 4) or

clearly labelled (in Fig. 5) any models that exhibited unusual divergent transitions (> 40 or > 1% of all MCMC runs).

Finally, there is a discrepancy in data resolution that requires explanation. The manuscript states that the occurrence information has an accuracy of <10 km (line 410) and the original climatic information is at 1 km resolution (line 416). The authors then state they aggregated the climatic data to 25 km (line 418). The rationale for choosing to aggregate the high-resolution climatic data to a much coarser resolution, thereby losing informative variance, must be justified.

Thanks for pointing out this discrepancy. Based on your comment, we noticed a small error in the code where we were thinning occurrence data using a 25 km grid instead of a 0.25° grid matching the climate data resolution. We have corrected this and now use the same 0.25° grid for both occurrence data thinning and climate data resampling. Furthermore, we corrected these inconsistencies in the manuscript.

As a separate step, we filtered out occurrences with a GPS uncertainty (which is sometimes provided by GBIF data in meters) of > 10 km for data quality control. We decided on +/- 10 km as a reasonable threshold since most of our occurrence data is centered around 45° latitude, where 0.25° is approximately equal to 20 km longitude. This ensured that the retained occurrence points would probably fall within the correct 0.25° climate grid cell.

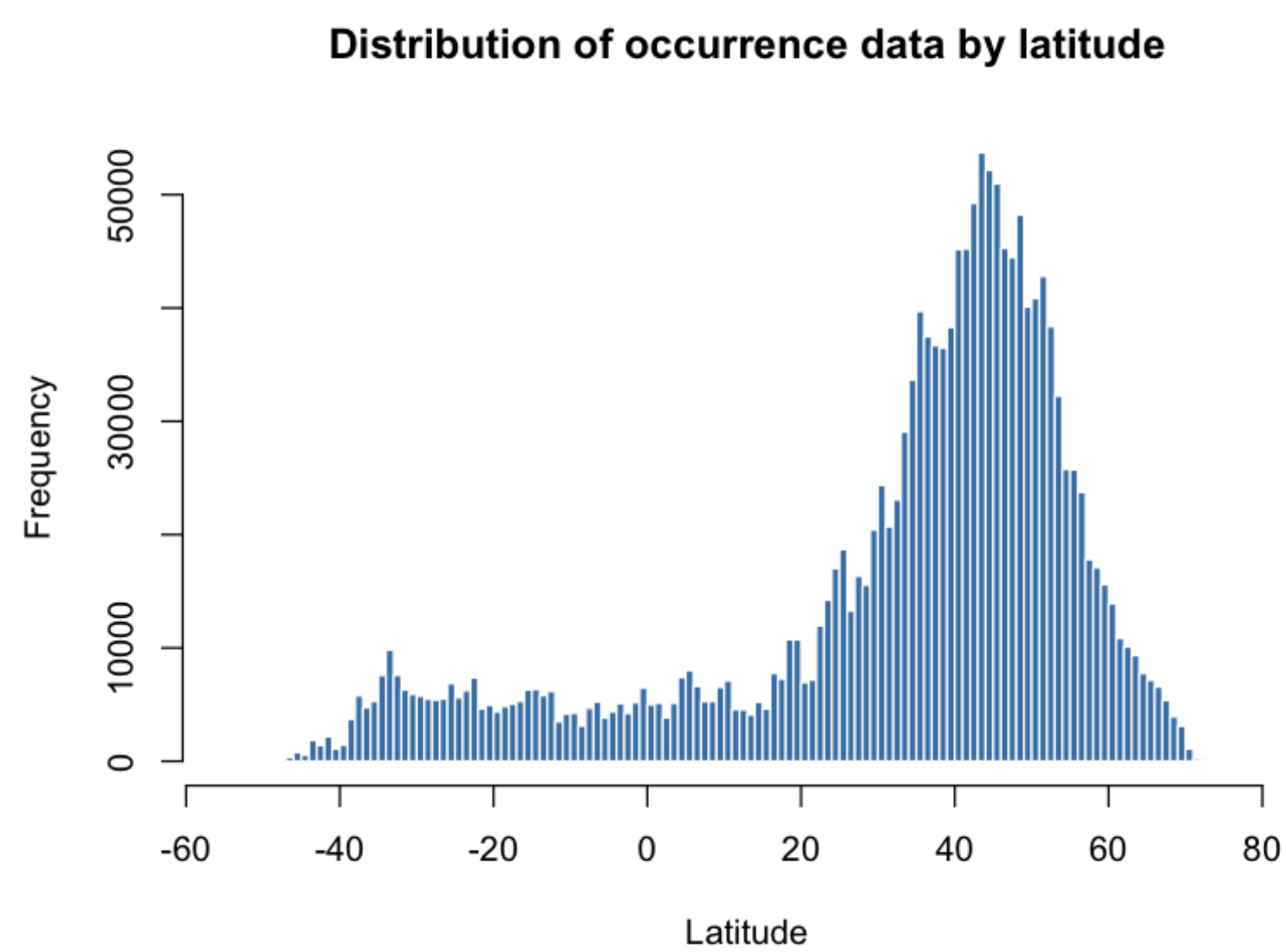

Furthermore, our rationale for aggregating the 30 arc second (~ 1 km) CHELSA data to 0.25° spatial resolution is expanded upon below:

Most importantly, we wanted to avoid the problem of false precision, which is common when combining raster climate data with species occurrence points. We cannot extract 30 arc second (~1 km) resolution climate data for occurrence points with up to 10 km spatial uncertainty. Unfortunately, reducing the spatial uncertainty threshold was also not an option (at least 500 m to accommodate CHELSA data at its

native resolution of ~ 1 km). This is because many valuable occurrence datasets for tree species are based on gridded national forest inventories, such as EU-Forest from Mauri et al. (2017), with inherent resolutions of 1 – 10 km. Herbarium samples, which do not have exact geolocations, could also be utilized (see Smith et al., 2023). Aggregating to 0.25° allows us to accommodate this uncertainty and utilize as many datasets within GBIF as possible.

Moreover, spatially thinning occurrence data to a 0.25° grid also helps reduce spatial autocorrelation and geographic sampling bias, like the clustering of records near major settlements. Since we also model niches at the genus level, we want to capture broad macroecological patterns, rather than overfitting individual species to local-scale climate.

Lastly, the computational cost of model fitting was also a practical consideration. Fitting all niche models currently takes approximately one week on a powerful workstation. Increasing the resolution would make the analyses computationally unfeasible while simultaneously increasing noise and excluding many valuable gridded occurrence datasets that cannot be used at finer resolutions.

To improve clarity about these decisions, we have added a few lines in the Methods section explaining both the resolution choice and the GPS accuracy filter with an appropriate reference.

> NEW:
> "Climate data was resampled from 30" to a 0.25° spatial resolution to reduce sampling bias, spatial autocorrelation, as well as the computational cost of model fitting."
>
> "Setting the GPS uncertainty threshold to 10 km also allowed us to accommodate both gridded occurrence datasets like national forest inventories as well as herbarium samples without exact geolocations (Smith et al., 2023)."

Reviewer #2 (Remarks to the Author):

The authors tried to use the fossil dataset of tree genera from Western and Central Europe to analyze tree diversity changes during the Cenozoic. This is a very interesting and useful work, especially for their implications for the adaptation of the region's ecosystems to ongoing climate change. The authors given the dynamic change trend of tree genus richness, and most of the tree genus losses are related to heat-tolerant lineages. Finally, this work provided several ways that inform forest management to increase regional tree diversity and enhance forest resilience under climate change based on paleoecological data. I have several concerns for this work.

We thank Reviewer #2 for the feedback and positive assessment of our manuscript!

First, I see the authors compiled a dataset of macrofossil and fossil pollen occurrences of tree genera. But I haven't seen where is the dataset. This is very important for this work.

All data used was made available in a Zenodo repository (https://doi.org/10.5281/zenodo.17079667). However, based on the reviewer's comments, we opted to develop an interactive dashboard hosted on GitHub Pages linked to the paper's repository that includes a navigable table of our dataset that can be easily searched and downloaded.

This is available at the following link: https://vfwilkens.github.io/PaleoTrees-WCE/#data. We think this substantially increases the accessibility to the underlying dataset.

Second, the analysis only focus on the tree genera in Western and Central Europe, why not include Northern Europe and Southern Europe in the analysis?

Thanks for this question. We focused our analyses specifically on Western and Central Europe for several reasons:

1) Western and Central Europe comprises the core of the European temperate zone and contains some of the most managed forest ecosystems in the world today (e.g., widespread Norway spruce monocultures). Here, the problem of forest reorganization is the most urgent due to ongoing changes in biotic and abiotic disturbance regimes as well as forecasted climatic shifts (see Wessely et al., 2024; Seidl et al., 2020; Seidl & Turner, 2022).
2) Northern Europe (i.e., Scandinavia) contains comparatively few fossil findings and would thus not add any helpful information to our study. At the same time, including Northern Europe would also expand the scope of the study into the current boreal zone, which we aimed to avoid as these forest ecosystems are distinct from temperate forests and would complicate interpretation of our results.
3) The paleoecological history of Western and Central Europe is fundamentally distinct from that of Southern Europe. Southern Europe is situated south of major east-west biogeographic barriers like the European Alps, the Pyrenees, and the Carpathian Mountains. These barriers are considered to have restricted range shifts of trees during Plio-Pleistocene climatic changes (Reid, 1935; Huntley, 1993), leading to dramatic losses in regional tree diversity that we observe today. Southern Europe acted as a major refugium for many genera (such as *Castanea*, *Platanus*, *Aesculus*, *Juglans*) that went regionally extinct in Western and Central Europe but persisted in the Mediterranean basin.

We briefly state these justifications at the end of the 5th paragraph in the Methods section:

> "This study area was chosen because it forms the core of the European temperate zone, contains heavily managed forest ecosystems, and has been disproportionally impacted by paleoclimatic changes due to its geographic configuration."

Third, many genera have very large latitude span, while the species occurrence data was downloaded from GBIF for all tree species belonging to genera in the final fossil occurrence dataset. Their climate niche evolution maybe change to diversity along their dispersal and new habitat. Thus, the reasonability to use

the current climate data of all species in the genera is open to discussion.

Thanks for this comment! This is a very good point. We agree that genera with large latitudinal spans may undergo climate niche evolution as species disperse into new habitats. We already discuss the implications of this to some extent in the 4th paragraph of the Discussion.

To address this concern more explicitly, we rely on the well-established assumption of phylogenetic niche conservatism (PNC). Existing literature provides a strong foundation for assuming PNC at the genus level in trees (Prinzing, 2001; Svenning, 2003; Crisp & Cook 2012; Hardy et al., 2012; Hawkins et al., 2013; Eiserhardt et al., 2015; Vetaas et al., 2018, etc.). Furthermore, using modern climate data and species occurrences to reconstruct paleoclimatic niches and paleoclimate itself is a widely accepted methodology, though we acknowledge its inherent assumptions and limitations (e.g., Gibson et al., 2022).

Moreover, our application of PGLMMs is also very robust against this concern. We do not treat modern species as independent data points. Instead, our niche models incorporate phylogenies to explicitly account for the covariance structure among species based on their shared evolutionary history. As a result, individual species that have dispersed into novel environments and evolved highly derived niches cannot overinfluence estimates of genus-level averages, because these are regulated by evolutionary distances. We have added some additional text to the manuscript Discussion to further clarify this justification.

> REVISED:
> "Next, we developed genus-level niche models to link paleoecological findings with contemporary biodiversity research and forest management (Smith et al., 2019). These models leverage phylogenetic relationships within genera to account for the non-independence of occurrences and species as well as compensate for species-level differences in occurrence data availability. A necessary assumption underlying this approach is that closely related species will tend to occupy similar environmental niches (i.e., phylogenetic niche conservatism). This assumption is well-founded (e.g., Prinzing, 2001; Svenning, 2003; Crisp & Cook 2012; Hardy et al., 2012; Hawkins et al., 2013; Eiserhardt et al., 2015; Vetaas et al., 2018) and is used frequently to reconstruct paleoclimate (e.g., Elias, 1997; Croft et al., 2018; Gibson et al., 2022; Xu et al., 2025). If phylogenies have not been adequately resolved, estimates for individual genera could be biased, but this would not impact the overall interpretation of our findings."

Reviewer #3 (Remarks to the Author):

The paper is quite innovative: it presents a complex processing of Cenozoic record of 200 modern tree genera in order to depict some climate changes in past as well as to give some predictions for forest management within the warming trends in future. I consider it as fully acceptable for being published in Nature Communications !

I put all my comments / questions or even corrections directly in the annotated version of the manuscript, in the attached file called "652672_0_art_file_294013_t2bym8-js.pdf", so only general points:

We thank Reviewer #3 for the considerable time and effort put into reviewing our manuscript, it is greatly appreciated! We respond to the general comments below and provide additional responses to all in-text comments afterwards.

I must openly admit that the statistical part concerning the correct application of R statistical software, concept of PGLMMs and related mathematical issues are rather deep to me, and this part, which very important, must surely by review by someday (skilled statistician), who is familiar with those problems in order to check manuscript independently !

On the other hand, I could give some (I hope constructive) comments relative to the palaeobotanical frame of the manuscript. Here, I think as very preliminary and also most important step is the correct choice of 200 modern genera and their distribution in time and space (WCE). As I noticed in my detailed review, I consider PBDB database in its present state as very incomplete so useless… In other words, today, its application can be very misleading – in this respect, the most important part of the manuscript to me as a palaeobotanist is about a correct application of the references present in the columns "Notes" and "References" in the file called "literature_records_28_01_25.lsx" in Supplementary material - unfortunately, many important pieces of information is relatively hidden there…

Thanks for this comment! We certainly agree with the assessment that PBDB is an incomplete database, which is why we have taken steps to account for this in our methods. For one, we do not rely only on PBDB to compile our fossil occurrence dataset. In addition, we have sorted through hundreds of peer-reviewed publications to supplement the data obtained from PBDB. This includes many global and regional palaeobotanical studies as well as larger works like academic books (from Mai, Lang, etc.). From these additional sources, we obtain hundreds of fossil occurrences that are otherwise missing in PBDB. The result is a dataset that provides a state-of-the-art synthesis of available fossil occurrences of tree genera for our study region and time span. We make this dataset readily available now also via an interactive dashboard (https://vfwilkens.github.io/PaleoTrees-WCE/#data) that enables easy navigation and downloading. Furthermore, we have made sure to distinguish occurrences that are obtained from PBDB and those that were obtained only via literature search. We hope this addresses the reviewer's concerns regarding the dataset being hidden in the supplementary material.

> REVISED:
> "PBDB is a paleontological database that was chosen as the basis for our fossil dataset because it is curated and contains standardized fossil occurrences across all geological ages. However, because PBDB is incomplete, database occurrences from PBDB were supplemented with additional records identified through literature searches (search string: e.g., "Quercus" AND "fossil") in Web of Science and Google Scholar."

Generally, there are taxa, which are rather general and cumulative as Ficus (can be related to other types), or Laurus (can you distinguish it from Laurophyllum or other members of Lauraceae, and what about Laurinoxylon and the fossil woods in general?) or others, which can be similar based on foliage to modern

ones (e.g., Cercidiphylum, Trigonobalanus), but they in fact represent extinct types as extinct Cercidiphyllum-like plants in Paleocene/Eocene or extinct Trigonobalanopsis/Eotrigonobalanus. Generally, many Paleocene sites in WCE were not systematically revised from "classical" times and there are surely many extinct genera (or stem group member?) as well as in Eocene sites, which are however better known. To sum up, I would put once again a special care to the table "literature_records_28_01_25.lsx" as this is the point of departure for any subsequent analysis and correct results !

We fully agree that taxonomic ambiguity, especially regarding so-called form taxa and fossil taxa is a significant challenge when working with palaeobotanical data. To avoid the problems you raise, we deliberately excluded all fossil or form-taxa (like *Laurophyllum* or *Laurinoxylon*) from our dataset and include only genera that have been taxonomically resolved to a modern genus. Excluding these unresolved and globally extinct taxa represents a conservative approach to our research question. Since these taxa are globally extinct, keeping them in the dataset would only inflate any estimates of tree diversity losses for Western and Central Europe. Genus-level niche estimation would also be biased, as we would be forced to use a nearest living relative approach leading to an overrepresentation of the more common form taxa and their relatives. Furthermore, because our study aims to link paleoecological filtering in deep time directly to modern challenges in forest reorganization, it is conceptually necessary to focus on lineages that are not globally extinct.

Nevertheless, we acknowledge your concerns regarding taxa that can be morphologically broad or "cumulative" e.g., *Ficus* or *Laurus*. We made sure these genera were labelled clearly in Fig. 4a and included additional explanations in the Methods and Discussion regarding the points raised.

> NEW:
> "Form genera and other globally extinct taxa were also excluded, as these cannot be reliably resolved to a modern genus. Furthermore, since we aim to link paleoecological filtering in deep time to current challenges in forest reorganization, it was conceptually necessary to focus on lineages that are not globally extinct."

> NEW:
> "Form genera and other globally extinct taxa (e.g., *Laurophyllum*) were excluded from our analysis (see Methods). Consequently, estimated tree diversity losses likely represent a lower bound to true losses. If these unresolved lineages could be accounted for, the degree of regional extinctions would probably be even higher and could also shift drivers of early Cenozoic beta diversity from nestedness to turnover (Kvaček, 2010)."

More details can be found in the attached files as commentaries, I also add two examples of comparison between PBDB and the "real" fossil record in order to demonstrate incompleteness of that database. However, I am really happy to see such an innovative and complex paper dealing with Central European Cenozoic Palaeobotany !

Thank you very much for the examples! Indeed, these demonstrate major discrepancies between PBDB and the underlying literature. We checked to see if these occurrences were missing from our dataset and found that they had already been included through literature searches. We hope this assures the reviewer that we adequately accounted for the incompleteness of PBDB in our study.

Reviewer #3 comments from the manuscript (in order as they appear):

Here, I would surely add this reference, which gives a complex overview of climate and vegetation history in the Cenozoic of the Central Europe, from late Eocene till early Pleistocene : http://www.geology.cz/bulletin/contents/art1553

Good suggestion. The reference has been added.

I suppose, you mean "extirpation" as the loss of a taxon in a specific area ? see Nature through Time (2020): https://link.springer.com/book/10.1007/978-3-030-35058-1

Indeed, "extirpations" are often used as a synonym for regional extinctions. However, for a broader interdisciplinary audience in *Nature Communications*, we believe "regional extinctions" is easier to understand. We added "(i.e., extirpations)" after the first mention of regional extinctions for clarity in the Introduction.

> REVISED:
> "However, over the Pleistocene, repeated glaciations triggered regional extinctions (i.e., extirpations) of many temperate tree taxa that persisted in other parts of the Holarctic realm (Ricklefs & He, 2016)"

Maybe, add some better and more complex explication ?

We added a source here for the "option-space" terminology (Wessely et al., 2024). We hope this helps clear up any confusion.

I think it is very important to understand correctly this crucial and fundamental concept to follow your text, mainly for somebody as me, who is not familiar with PGLMMs... That's why, I think, would be suitable to explain it even here, or surely before line 430 !

Thanks for the feedback. Unfortunately, we do not think that we can go much more into detail at the end of the Introduction regarding the methods used. We already dedicate Fig. 1, its caption, as well as the last paragraph of the Introduction to doing so. For this reason, we think the current description is sufficient but will defer to the editor's opinion on this point. We also added references and rewrote part of the Methods regarding the niche modelling approach in response to a comment above, which may help to clarify this point as well.

Generally, I don't understand why the methods are explained only at the end of the text ?

The Methods are located at the end of the manuscript as this is the official format for studies published in *Nature Communications*.

You mean WCE ?

Added for clarification.

Maybe explain here as it is the first occurrence in the text, and not only in lines 182-183

We define the "Holocene regional tree flora" already in this sentence. We added quotation marks around it to make it clear that this sentence defines this terminology.

> REVISED:
> "Additionally, we fitted niche models for all extant genera using only species native to Western and Central Europe, which we refer to as the "Holocene regional tree flora"."

I am convinced that the PBDB an its present stage is very "shaky", so useless, and it can be very misleading regarding the exact depiction of total geographical and time range of taxa...

See our response above. We agree, but by supplementing PBDB occurrences with other literature sources, we largely overcome the incompleteness of the database.

As an example, I send attached the PBDB print-screens of two fossil species + their time/area range based on two publications by Kvaček

See our response above regarding the provided examples. These occurrences were already included in our dataset via literature searches.

What does it mean in the picture left down those "715 fossil occurrences" ?

This refers to the total number of fossil occurrences in the final dataset after filtering and pre-processing, which were then used for further analyses (does not include Holocene occurrences since these were not obtained from fossils). We updated the figure as this number is now 711.

> REVISED:
> "**a**, 711 fossil occurrences of tree genera during the Cenozoic (66 Ma – present) from Western and Central Europe (including Austria, Belgium, Czechia, France, Germany, Ireland, Luxembourg, Netherlands, Poland, Switzerland, and United Kingdom) were compiled using the Paleobiology Database (PBDB) and literature."

see my comment above concerning PGLMMs

We address this above. In summary, we feel like the current description of Methods at the end of the Introduction is adequate, especially given the expanded explanation now in the Methods, but would defer to the editor's opinion if we should expand this part still.

To me, Miocene as well as Eocene represent very large time slices (duration about 18 and 22 Ma respectively), comparing to other Epochs, or even Ages in Pleistocene...

Thanks for the comment, but we are not sure how to respond to this. We show tree genus richness for the entire Pleistocene in Fig. 2 (the dotted horizontal lines). Improving the resolution of the Pleistocene by using interglacial periods and temperate phases will inherently lead to smaller time slices.
Moreover, Miocene has roughly in the middle the last warm peak in Cenozoic, i.e., MCO between ca 17 and 15 Ma, so here it would be more suitable/correct to characterize the floras before-, during- and after-

We fully agree that the MCO was likely a critical climatic event that also shaped the Cenozoic tree flora. However, subdividing the Miocene into pre-MCO, MCO, and post-MCO bins requires fossil occurrence data with very tight chronological control (e.g., radiometric dating or high-resolution biostratigraphy). A substantial portion of the fossil record in Europe is broadly dated only to the "Miocene" or to coarser sub-epochs (Early/Middle/Late). Subdividing the Miocene would force us to either discard a large portion our data or risk assigning occurrences to the wrong time bin. Ultimately, this would only amplify taphonomic biases, since instead of one complete picture of the Miocene, we will have three or more smaller time bins that are mostly incomplete. Therefore, utilizing the broader epoch-level chronology provides the best possible framework for assessing if a genus was present or not. Lastly, we note that aggregating/synthesizing existing studies to a single dataset with epoch-level temporal resolution, as we do here, is already a novel contribution.

Btw, it is not a problem if you define for your model and subsequent interpretations uneven time slices ??

This is not an issue as time bins or time bin durations are not included in our niche models. We fit the niche models only using species occurrences, phylogenies, sampling bias weights, and current climate data.

Maybe replace by "conifers and ginkgo" to be more specific ?

Added this to the start of Results.

REVISED:
"We started by summarizing major trends in tree genus richness during the Cenozoic based on fossil occurrences, highlighting differences between angiosperms and gymnosperms (i.e., conifers and Gingko)."

How this nestedness-dominated peak can be changed if we include also extinct genera ??

(in my opinion, it would rather be a turnover)
In fact, in Paleocene, there are many extinct genera still present, and these can in reality represent the stem-lineage members of taxa, being only hardly recognized without co-occurring reproductive organs. Moreover, Paleocene site in WSE are rather few, and they are generally not revised from "classical" times, see here :
http://www.geology.cz/bulletin/contents/art1146

Great question! We agree with your assessment. The nestedness peak during the Paleocene-Eocene transition is driven by the apparent emergence of 78 genera in the fossil record of WCE. If we included extinct genera and form-genera from the Paleocene, this nestedness signal would probably shift toward turnover.

We added a justification for excluding form genera and fossil taxa from our dataset in the Methods as well as discussion of the implications of this choice in the Discussion.

> NEW:
> "Form genera and other globally extinct taxa were also excluded, as these cannot be reliably resolved to a modern genus. Furthermore, since we aim to link paleoecological filtering in deep time to current challenges in forest reorganization, it was conceptually necessary to focus on lineages that are not globally extinct."
>
> NEW:
> "Form genera and other globally extinct taxa (e.g., *Laurophyllum*) were excluded from our analysis (see Methods). Consequently, estimated tree diversity losses likely represent a lower bound to true losses. If these unresolved lineages could be accounted for, the degree of regional extinctions would probably be even higher and could also shift drivers of early Cenozoic beta diversity from nestedness to turnover (Kvaček, 2010)."

I suppose you mean in general ? e.g., there are only 8 species in Syringa, but I see 16 species only in China here: http://www.efloras.org/florataxon.aspx?flora_id=2&taxon_id=132143 ...so check it once more !

Thanks for pointing this out. We used the Botanic Gardens Conservation International (BGCI) GlobalTreeSearch database as our authority for how many extant tree species are recognized within each genus globally. This database is compiled by experts and is also the basis for the State of the World's Trees report. Note that we only counted species here that are considered by BGCI to be tree species and other life forms (shrubs, herbs, etc.) within each genus were not included. This explains the discrepancy with the linked eflora checklist from China. With respect to the goal of our study, which is to link paleoecological changes to current challenges in forest reorganization, it makes sense to focus exclusively on tree species.

Why ? Maybe put here some provisional explication

A provisional explanation here would not be appropriate in the Results. However, upon consideration, we decided to remove this part of the sentence since it is not directly relevant to our research questions and study.

I suppose you are still using PGLMMs concept and only the central points of the circles are important here and they represent the genus-level averages (β), is that right ?

That's correct.

or Gelman-Rubin ?

Fixed, thanks!

I suppose from total 200 genera + several others in the the Holocene regional tree flora ?
So, ho many in total in the original pool ??

The distribution of the categories under the fitted models was:
Extant genera: 36
Holocene regional tree flora: 33
Regionally extinct genera: 163

We tried to fit 237 niche models in total (200 regionally extinct genera, 37 extant genera and 37 genera from the Holocene regional tree flora). However, only 232 models were fit due to five cases where the corresponding species pool contained no tree species, according to BGCI and the underlying IUCN definition (like *Myrica,* which is native to WCE but has a single native WCE species, *Myrica gale* L., which is not classified as a tree). We added an explanation in the Methods:

> NEW:
> "We distinguished between regionally extinct (n = 163) and extant genera (n = 37) in our analysis. Additionally, for each extant genus, we fitted PGLMMs using only the species native to Western and Central Europe, which we refer to as the Holocene regional tree flora (n = 37). We attempted to fit all 237 PGLMMs, but there were five cases where it was not possible fit a model because the corresponding species pool contained no tree species. This resulted in a final total of 232 models of both climatic centers and thermal limits, respectively."

this can only be seen with a microscope, generally the Fig. 5 has many pieces of information and the letters/names are too small to be visible, only in digital format, in high magnification...

Thanks for the feedback. We adjusted this figure to comply with formatting standards for *Nature Communications* and now use 5 pt font for the genus labels. In general, the figure can also be understood without having to read the genus labels, which provide more information in the digital version. However, this is a formatting or stylistic issue so we would defer to the editor's opinion about this.

REVISED:

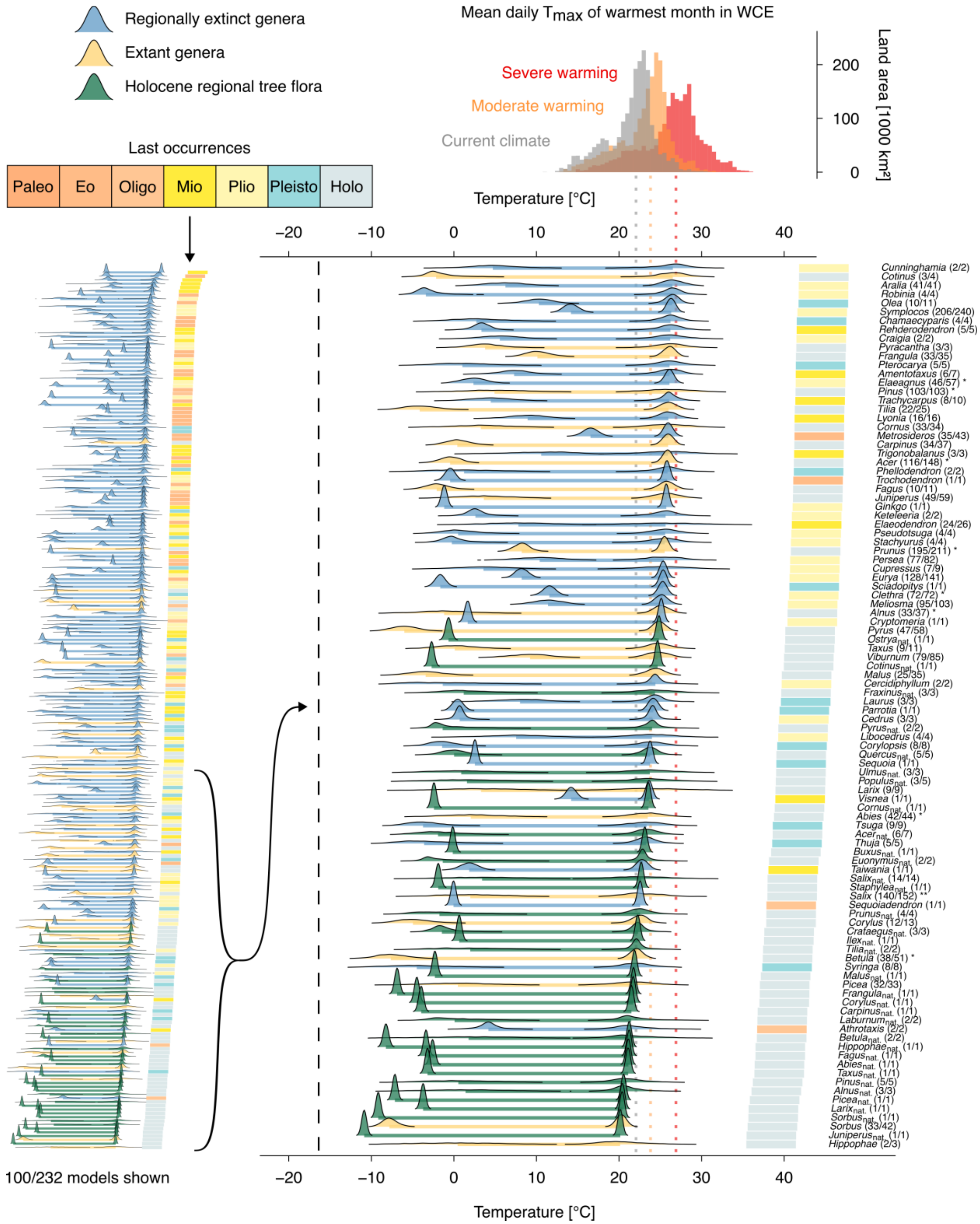

...just two questions here :

1) what about precipitation here as this can also influence the resilience oh extant genera in future climate scenarios ?

We agree that precipitation is important, which is why we include annual precipitation in our models of niche centers (Fig. 4). However, precipitation cannot be treated analogously to thermal limits with genus-level averages of maximums and minimums to be estimated. Drought impacts depend on soil moisture, which is dependent not only on soil properties, but also on so-called antecedent precipitation. Furthermore, forecasted precipitation changes across Europe are heterogenous, with northern regions expected to see increases while southern regions will see decreases. Seasonality of precipitation is also expected to change. See also Fig. 4, in which annual precipitation under SSP-370 and SSP-585 will not really change at all. For these reasons, defining meaningful upper and lower precipitation boundaries for genera is considerably less straightforward than for temperature. To answer your question directly, we don't think it makes sense to extend the niche limit approach to precipitation "limits" in our study.

2) your interval here are really maximal to include the very limits, within which the tree can +-survive, but are there some included intervals in which the trees can really thrive ?
(I think such intervals would be more suitable for any forest management in future climate scenarios...)

First, we want to re-iterate that genus-level niche limits should not be interpreted as strict physiological thresholds for individual species. We address this already in the Discussion, but it is very possible that some species will deviate significantly from genus-level averages in their limits or optima. Nevertheless, modeling at the genus level provides a practical and robust framework for comparing taxa above the species level directly (see Smith et al., 2019), while mitigating issues such as phylogenetic non-independence among species and sampling biases in occurrence data.

Identifying the conditions under which a species thrives and not just survives is a key question for forest management. To identify and model these optimal zones, though, we would need high-resolution performance data such as abundance, growth rates, or tree ring widths. However, these are not available at anything close to the scale needed for our study. Therefore, in our study, we can only focus on limits for now.

too long...
see my comment above, line 110

We understand your concern. Please see our response above regarding splitting up the Miocene relative to the MCO.

Sorry, I do not understand the meaning... Can you explain it, even in your text ?

This section was re-worded in response to a different comment. Please check again. Otherwise, we also improved the explanation in the Methods, including additional references.

To me, as a palaeobotanist, this statement is very very crucial as all your presumptions are base on "Uniformitarian Principle", i.e., fossil plants (genera) had the same climatic requirement as their today's nearest living relatives (genera), but this is not always true, and we know that already...
e.g., see here :
https://doi.org/10.1016/j.palaeo.2007.03.032

Yes, this principle (i.e., niche conservatism) is certainly a key assumption is our study but is also very well-established. There are limitations and it is important to be aware of them and how they could impact results. However, the modeling approach we use helps us account for this. See our response above to Reviewer #2 regarding PNC. We discuss this also in the Discussion:

> REVISED:
>
> "Next, we developed genus-level niche models to link paleoecological findings with contemporary biodiversity research and forest management (Smith et al., 2019). These models leverage phylogenetic relationships within genera to account for the non-independence of occurrences and species as well as compensate for species-level differences in occurrence data availability. A necessary assumption underlying this approach is that closely related species will tend to occupy similar environmental niches (i.e., phylogenetic niche conservatism). This assumption is well-founded (e.g., Prinzing, 2001; Svenning, 2003; Crisp & Cook 2012; Hardy et al., 2012; Hawkins et al., 2013; Eiserhardt et al., 2015; Vetaas et al., 2018) and is used frequently to reconstruct paleoclimate (e.g., Elias, 1997; Croft et al., 2018; Gibson et al., 2022; Xu et al., 2025). If phylogenies have not been adequately resolved, estimates for individual genera could be biased, but this would not impact the overall interpretation of our findings."

Personally, it is strange to see "Methods" at the end of a paper ??? I would surely move it at the beginning, at least before "Results" and "Discussion" !

This is the official format for *Nature Communications*.

On the other hand, I completely miss part called "Conclusions" in your text, in which the main points would be summarised clearly, why is it not here ?
("Discussion" is not the same...

According to https://www.nature.com/documents/ncomms-formatting-instructions.pdf we cannot have a separate Conclusion section. However, the last paragraph of the Discussion is effectively a conclusion.

see my comments above as well as in "general" review...
see papers by Kvacek and other Tertiary palaeobotanist as Manchester, Kovar-Eder etc, e.g. for Plio-Pleistocene of Czechia here: https://doi.org/10.1016/j.palaeo.2015.09.038

Thanks for the suggestions. See also above our general response regarding the incompleteness of PBDB. Numerous studies by these authors have already been accounted for in our dataset. We checked the suggested study that was linked, but it did not add any new fossil occurrences.

REVIEWER COMMENTS

Reviewer #1 (Remarks to the Author):

The authors have revised the manuscript and answered the questions raised after first revision, which is highly appreciated, resulting in an improved version of their work. Although the authors have resolved all questions and concerns by amplifying the methodology and discussion sections, there are still a few considerations still worthy to highlight.

1.Regarding the choice of PBDB:

The authors state in their reply that: "In general, fossil pollen records require very careful quality control due to common taphonomic issues, such as older grains being deposited into younger sediments leading to anachronistic "false occurrences" of some taxa. […]. Therefore, it did not make sense to query Neotoma/EDP directly because we assumed that any relevant studies included in these databases would probably appear anyway during literature searches".

Also they add that "while Neotoma does also contain plant macrofossil occurrences, we checked these and concluded that these occurrences were either redundant or had already been found during our literature search. Thus, they did not add any new information to our dataset".

First, there appears to be a discrepancy between the authors' response and the text; while the former implies the study relies solely on macrofossils, lines 338–339 indicate the inclusion of fossil pollen. Aside from this specific mention, there are no other references to pollen or macro-remain records throughout the manuscript.

Second, based on the authors' response, it appears that pollen records were excluded to avoid anachronistic 'false occurrences' arising from taphonomic issues such as redeposition. However, this stance constitutes a significant critique of a vast body of palynological research spanning the Quaternary and Tertiary. It is worth noting that redeposited grains are often distinguishable from contemporaneous pollen by their state of preservation. While pollen data has inherent limitations, it remains a fundamental source of evidence for reconstructing European paleo-flora.

I recommend clarifying if the analysis excludes pollen records. If so, please use the term 'macrofossil' instead of the more general 'fossil' in the appropriate sections (for instance, lines 22, 337, and 364) to more accurately reflect the data source.

Thanks for giving us the opportunity to clarify this. We understand how this probably got confusing in the response and manuscript text.

To be clear, our dataset does include fossil pollen occurrences (for example: fossil pollen findings of *Rehderodendron* from Hofmann & Zhao (2022) dated to the Eocene and Miocene). However, individual findings of fossil pollen (often associated with fruits or other plant structures) must be distinguished from fossil pollen records obtained from sediment cores (like in bogs or lakes). While fossil pollen itself can certainly be dated back to millions of years or more, high-resolution dating of fossil pollen records requires continuous stratigraphic sequences. Such sediment cores in Europe rarely extend back much further than 100 kya due to recurrent resurfacing of landscapes by glaciers during the Pleistocene. Neotoma and EDP are databases that contain mostly fossil pollen records obtained from such sediment cores and therefore cannot contribute meaningfully to our long-term fossil occurrence dataset (see below Fig. 2b from Flantua et al., 2023, which provides a temporal overview of pollen records in the Neotoma database). This is why we chose not to query these databases (as we explained in the lines you quoted), but why our dataset still includes some fossil pollen occurrences sourced from literature and even a few from PBDB.

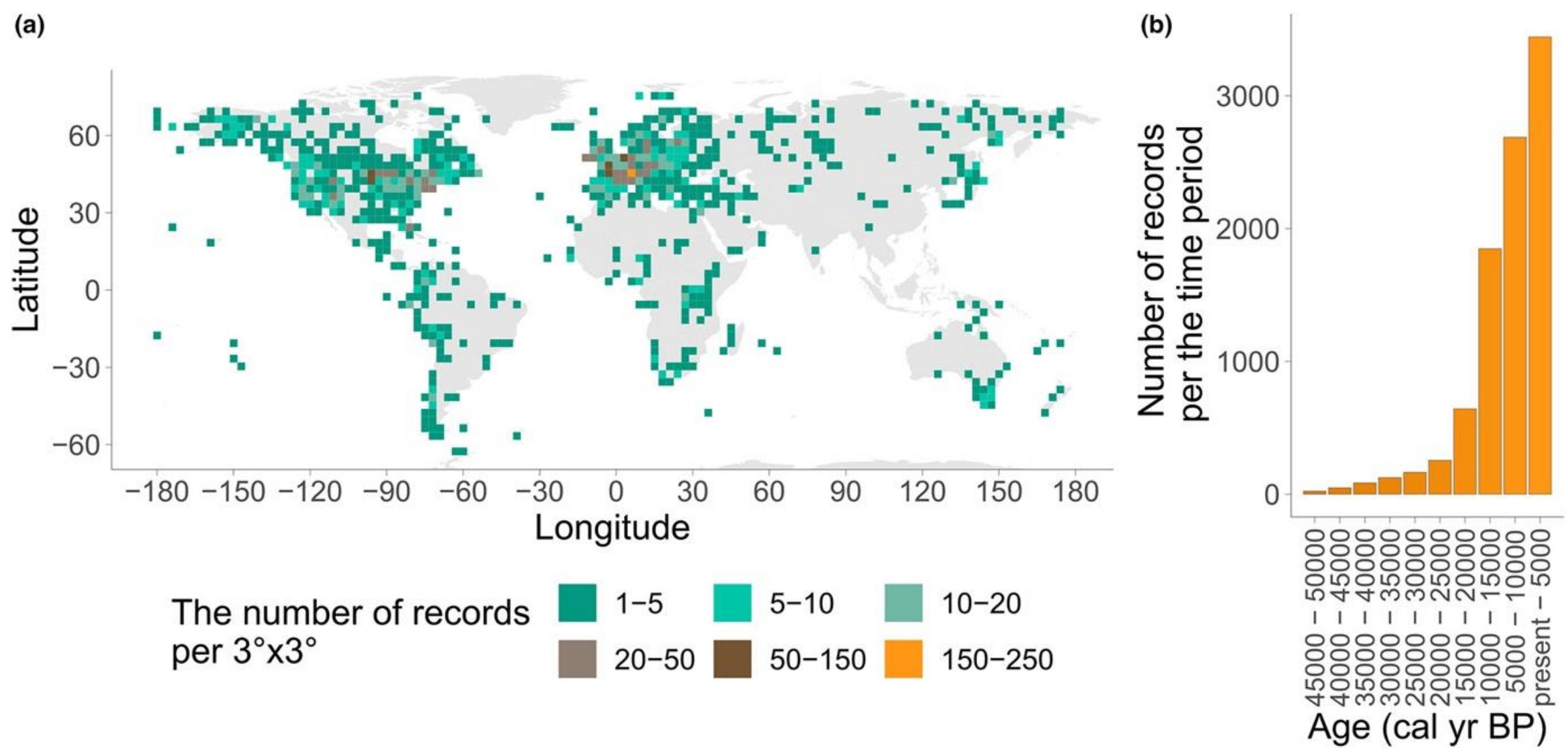

Figure from Flantua et al., 2023: Spatial and temporal overview of pollen records in the Neotoma database

Flantua, S. G., Mottl, O., Felde, V. A., Bhatta, K. P., Birks, H. H., Grytnes, J. A., ... & Birks, H. J. B. (2023). A guide to the processing and standardization of global palaeoecological data for large-scale syntheses using fossil pollen. *Global Ecology and Biogeography*, *32*(8), 1377-1394.

Since the differentiation of “fossil occurrences” into “macrofossil and fossil pollen occurrences” does not appear elsewhere but at the beginning of the Methods and thus serves no purpose, we decided to simplify it to just “fossil occurrences” to reduce potential confusion about what pollen data were used or not.

> REVISED:
> “To analyze how tree diversity changed throughout the Cenozoic era in Western and Central Europe, we first compiled a dataset of **fossil occurrences** of tree genera.”

Lastly, it was not our intention to criticize palynological research. However, aside from the temporal mismatch between our dataset and most pollen records in Neotoma/EDP, there are also widely recognized practical considerations when working with pollen records that led us to decide against querying these databases in our case (see also Flantua et al., 2023). This is what we intended to explain in the previous response.

2. Regarding the choice of exclusion of Pleistocene occurrences from PBDB:
Authors answer to the raised question: "While PBDB is a great database for deep time occurrences of plant macrofossils, we opted to exclude Pleistocene (and Holocene) occurrences from our query due to issues with chronological precision. In our study, we represent the Pleistocene as five separate time bins based on the chronology from Lang (1994). However, the coarse epoch-level dating in PBDB (e.g., “Pleistocene” or “Late Pleistocene”) was not precise enough to be able to reliably assign occurrences to a specific interglacial period or temperate phase. Therefore, we decided to compile the Pleistocene portion of our dataset exclusively from literature and not from PBDB".

In light of this response, the previously raised question regarding the exclusion of databases such as Neotoma or the European Pollen Database (EPD) becomes even more pertinent. These sources provide a vast number of Quaternary records with greater chronological precision than those typically found in Tertiary studies. If pollen data were indeed utilized, I strongly suggest including a table of the sites and their corresponding references as supplementary material.

We think this point has already been addressed with our response above. While pollen records from Neotoma/EDP do offer higher temporal precision (such as 100 or 1000 year time bins), the disadvantage is that the maximum temporal extent of this data is much shallower (generally limited to after the Last Interglacial or < 100 kya as shown above). Therefore, we did not include pollen records from Neotoma/EDP in our dataset (again, not to be confused with individual findings of fossil pollen).

Furthermore, we do not believe it is sensible to include a supplementary table of individual fossil pollen occurrences that were used, as this would have the effect of giving this data some special status compared to other fossil data. Since the underlying fossil dataset itself and all sources used are already very accessible, we do not see the need for such a table that singles out fossil pollen occurrences.

3.The treatment of Holocene fossil occurrences:
I previously expressed concern regarding the treatment of Holocene fossil occurrences as modern presence data rather than as part of the fossil record. The authors have clarified that they did not use any Holocene fossil occurrence data in their study, noting that such findings are rare and do not fully represent current native tree diversity in Europe. However, it is widely recognized that Holocene fossil records are significantly more abundant than those from the Pleistocene or Tertiary.

This is a fair point! To clarify, we intended to state that there are only a few Holocene-era fossil occurrences available in PBDB, not in general. This is probably a consequence of PBDB not being a very popular data repository for Holocene-era paleontology or palynology. We revised the relevant sentence in the manuscript to make this clear:

> REVISED:
> "Lastly, to determine the **natural** presence of genera in Western and Central Europe during the Holocene, **we did not use fossil occurrences due to the scarcity of findings in PBDB as well as the difficulty of excluding human influences.**"

The authors haver also answered that they utilized the World Checklist of Vascular Plants (WCVP) to establish a country-level checklist of native tree genera for Western and Central Europe. First, I have not found any published basis for this specific methodology and suggest adding the appropriate reference.

Using country-level native botanical checklists from WCVP for regional or global biogeographic analyses is common (e.g., Taylor et al., 2023; Qian et al., 2024; Bachman et al., 2024) and even in conjunction with fossil pollen data (e.g., Walentowitz et al., 2023) for determining native or non-native status of taxa on an island.

In WCVP, "native" status, which is determined by experts and database curators based on existing literature, is defined as:

> "…areas where the [the species in question] have been present since before the last Ice Age or arrived by natural colonization."

We added the above definition and reference to the Methods section and hope it satisfies your request:

> NEW:
> "Instead, we obtained a modern checklist of accepted native tree genera for Western and Central Europe using WCVP, accessed via the "rWCVP" R package[68]. **WCVP determines a species to be native to an area where "[it] has been present since before the last Ice Age or arrived by natural colonization"[69]**."

References:

Bachman, S. P., Brown, M. J., Leão, T. C., Nic Lughadha, E., & Walker, B. E. (2024). Extinction risk predictions for the world's flowering plants to support their conservation. *New Phytologist*, *242*(2), 797-808.

Qian, H., Qian, S., Zhang, J., & Kessler, M. (2024). Effects of climate and environmental heterogeneity on the phylogenetic structure of regional angiosperm floras worldwide. *Nature Communications*, *15*(1), 1079.

Taylor, A., Weigelt, P., Denelle, P., Cai, L., & Kreft, H. (2023). The contribution of plant life and growth forms to global gradients of vascular plant diversity. *New Phytologist*, *240*(4), 1548-1560.

Walentowitz, A., Lenzner, B., Essl, F., Strandberg, N., Castilla-Beltrán, A., Fernández-Palacios, J. M., ... & Nogué, S. (2023). Long-term trajectories of non-native vegetation on islands globally. *Ecology Letters*, *26*(5), 729-741.

Finally, I also suggest replacing the label 'Holocene' with a more precise term, such as 'W-C Europe Native Status,' to better reflect the nature of the data.

The question of how best to refer to this category is something we discussed for a long time, eventually leading us to settle on "Holocene regional tree flora". For one, we wanted to avoid including "native" directly in the label as it puts too much emphasis on native/non-native status (which tends to mean different things to different people) rather than the temporally and regionally defined category used in our analysis. Secondly, we also wanted to avoid introducing an additional acronym like "WCE" as this reduces readability throughout the text.

Therefore, we would respectfully prefer to keep "Holocene regional tree flora". However, we acknowledge that there are still some ambiguities in the text and made several revisions to better introduce these categories explicitly in the Introduction and captions of Figs. 4 and 5:

> REVISED:
> "These hierarchical models were used to estimate genus-level averages of niche parameters while incorporating species-level phylogenetic covariance and correcting for sampling bias. **We compared niche parameters among three analytical groups with respect to Western and Central Europe: (i) regionally extinct genera, (ii) extant genera, and (iii) the current option-space for forest reorganization, hereafter referred to as the "Holocene regional tree flora". For regionally extinct and extant genera, niche parameters were estimated using all available tree species within each genus globally. For the Holocene regional tree flora, we fitted separate niche models for each extant genus using only the native species pools of Western and Central Europe. We then examined all three groups in the context of current climate and future warming scenarios for Western and Central Europe.** With this analysis, we address the following question:"
>
> REVISED:
> "Fig. 4: Average locations in climate space of Cenozoic tree genera from Western and Central Europe. a, **Estimated climatic centers of 200 tree genera, comparing (i) regionally extinct genera (blue), (ii) extant genera (yellow), and (iii) the current option-space for forest reorganization, referred to as the Holocene regional tree flora (green). Climatic centers of regionally extinct and extant genera were estimated using all available tree species within each genus globally, whereas climatic centers of the Holocene regional tree flora were estimated for each extant**

genus using only the native species pools of Western and Central Europe (e.g., Abies$_{nat}$. represents *Abies alba* Mill.)."

REVISED:
"Fig. 5: Upper and lower thermal limits of Cenozoic tree genera from Western and Central Europe. **Posteriors of upper and lower thermal limits for 200 tree genera were estimated, comparing (i) regionally extinct genera (blue), (ii) extant genera (yellow), and (iii) the current option-space for forest reorganization, referred to as the Holocene regional tree flora (green). Thermal limits of regionally extinct and extant genera were estimated using all available tree species within each genus globally, whereas thermal limits of the Holocene regional tree flora were estimated for each extant genus using only the native species pools of Western and Central Europe."**

Reviewer #2 (Remarks to the Author):

The authors have improved the manuscript very well based on the three reviewers suggestions. I have no other questions for this paper.

Thank you!

Reviewer #3 (Remarks to the Author):

Dear Authors,
Congratulations once again on your innovative paper!
And thank you for taking all my comments into account in such detail; this is not common, and I appreciate it all the more.
I fully agree with your revisions and recommend publication in this form.
With warm regards,
Jakub Sakala
P.S.: Perhaps I would just put "form genera" in quotation marks, for example here:
Form genera and other globally extinct taxa (e.g., Laurophyllum)...
since these are now obsolete categories according to the ICBN; only "fossil taxa" is used exclusively, as nicely summarized here:
https://en.wikipedia.org/wiki/Form_classification#In_botany
However, I think you can leave it with the quotation marks, the people will understand :)

Thanks! We added the quotation marks.
